# Genome-wide 5-hydroxymethylcytosine (5hmC) emerges at early stage of *in vitro* differentiation of a putative hepatocyte progenitor

**DOI:** 10.1038/s41598-020-64700-2

**Published:** 2020-05-08

**Authors:** Jesús Rafael Rodríguez-Aguilera, Szilvia Ecsedi, Chloe Goldsmith, Marie-Pierre Cros, Mariana Domínguez-López, Nuria Guerrero-Celis, Rebeca Pérez-Cabeza de Vaca, Isabelle Chemin, Félix Recillas-Targa, Victoria Chagoya de Sánchez, Héctor Hernández-Vargas

**Affiliations:** 1 Department of Cellular Biology and Development, Instituto de Fisiología Celular, Universidad Nacional Autónoma de México (UNAM), Circuito Exterior s/n, Ciudad Universitaria, Coyoacán, 04510, Cd. Mx. Mexico; 2 Institute of Biology Valrose (iBV), The National Center for Scientific Research (CNRS) - National Institute of Health and Medical Research (Inserm), Université Côte d’Azur, Nice, France; 3 Department of Immunity, Virus and Inflammation. Cancer Research Centre of Lyon (CRCL), Inserm U 1052, CNRS UMR 5286, Université de Lyon, Centre Léon Bérard, 28 rue Laennec, 69373, Lyon, CEDEX 08 France; 4 Molecular Mechanisms and Biomarkers Group, International Agency for Research on Cancer (IARC), 150 Cours Albert Thomas, 69008 Lyon, France; 5 Division of Biomedical Research, Centro Médico Nacional “20 de noviembre”, ISSSTE, San Lorenzo 502, Benito Juárez, 03100, Cd. Mx. Mexico; 6 INSERM U1052, CNRS UMR5286, Centre de Recherche en Cancérologie de Lyon Université Claude Bernard, Lyon, France; 7 Department of Molecular Genetics, Instituto de Fisiología Celular, Universidad Nacional Autónoma de México (UNAM), Circuito Exterior s/n, Ciudad Universitaria, Coyoacán, 04510, Cd. Mx. Mexico; 8 Department of Translational Research and Innovation. Centre Léon Bérard, 28 rue Laennec, 69373, Lyon, CEDEX 08 France

**Keywords:** Cancer genomics, Epigenomics, DNA methylation

## Abstract

A basic question linked to differential patterns of gene expression is how cells reach different fates despite using the same DNA template. Since 5-hydroxymethylcytosine (5hmC) emerged as an intermediate metabolite in active DNA demethylation, there have been increasing efforts to elucidate its function as a stable modification of the genome, including a role in establishing such tissue-specific patterns of expression. Recently we described TET1-mediated enrichment of 5hmC on the promoter region of the master regulator of hepatocyte identity, *HNF4A*, which precedes differentiation of liver adult progenitor cells *in vitro*. Here, we studied the genome-wide distribution of 5hmC at early *in vitro* differentiation of human hepatocyte-like cells. We found a global increase in 5hmC as well as a drop in 5-methylcytosine after one week of *in vitro* differentiation from bipotent progenitors, at a time when the liver transcript program is already established. 5hmC was overall higher at the bodies of overexpressed genes. Furthermore, by modifying the metabolic environment, an adenosine derivative prevents 5hmC enrichment and impairs the acquisition of hepatic identity markers. These results suggest that 5hmC could be a marker of cell identity, as well as a useful biomarker in conditions associated with cell de-differentiation such as liver malignancies.

## Introduction

Dynamic changes in chromatin organization and DNA modifications participate in the establishment of cell identity in normal human biology and disease^1^. Epigenetic marks such as DNA methylation are characteristic of a particular cell type as they help define the associated transcriptome^2,3^. DNA methyl-transferases (DNMTs) establish 5-methylcytosine (5mC) from S-adenosylmethionine (SAM), the principal methylating agent in the body derived from the methionine cycle^4^. In humans, the liver is the organ with the highest turnover of SAM and metabolism of methionine^5^. It has been suggested, that adenosine could be able to modulate SAM methylation in the liver, promoting either the metabolic flow of methyl group transfer-reactions or their inhibition by inducing S-adenosylhomocysteine (SAH) accumulation^6^.

As opposed to DNMTs, Tet Methylcytosine Dioxygenases (TET) are involved in the oxidation of methylated cytosine in DNA forming 5-hydroxymethylcytosine (5hmC) and, together with the base excision DNA repair machinery, lead to active cytosine demethylation^7,8^. Several studies suggest that the dynamic distribution of 5hmC could be an acquired “imprint” of cell identity during adult progenitor cell differentiation in various tissues, including liver (reviewed in [^9^]). Indeed, apart from neurons and stem cells, the liver is relatively rich in 5hmC content compared to other adult organs^10^. Specific patterns of 5hmC have been shown in normal liver tissue^10–12^, and several studies have described an overall and gradual loss of 5hmC in liver cancer^13–17^ and other liver pathologies^18,19^. In addition, 5hmC is overall increased in human adult liver compared to fetal liver, with 5hmC occupancy overrepresented in genes involved in catabolic and metabolic processes^20^. Indeed, it was shown that the majority of age-related DNA methylation changes in mouse liver occur between postnatal days 5 and 20^21^, although that study could not distinguish between 5-methylcytosine (5mC) and 5hmC. On the other hand, *in vitro* approaches to hepatocyte differentiation using human mesenchymal stem cells showed that inhibiting DNA methylation could increase the efficacy of differentiation^22–24^. Moreover, a report described a transient accumulation of 5-carboxilcytosine, another intermediate on the process of active DNA demethylation, during differentiation of hiPSC to hepatocytes^25^. Although 5hmC distribution during adult progenitor cell differentiation has been assessed in several tissues (reviewed in [^9^]), there is a lack of information in liver. We described a specific shift in 5hmC at the *HNF4A locus* which occurs at one week of cell culture and that leads to unleashing hepatocyte differentiation^26^. However, there is no base-resolution genome-wide analysis of 5hmC during hepatocyte differentiation in a controlled system.

The capacity to modulate epigenetic modifications, offers an opportunity to assess how epigenomic changes could influence cell differentiation as well as to develop new strategies for the early prevention and treatment of diseases^27^. An adenosine derivative, IFC-305 (UNAM Patent 207422), is able to modulate SAM levels and regulates DNA methylation^28^, presenting hepatoprotective properties^29–34^. Therefore, this adenosine derivative could be a useful tool to understand how a metabolic environment could modify chromatin components during differentiation processes.

Here, we asked whether 5hmC is present and/or redistributed in the genome of differentiating hepatocytes. We describe 5hmC genomic enrichment and its relationship with gene expression. Moreover, we show how 5hmC accumulation and hepatocyte differentiation are impaired by perturbing the metabolic environment using IFC-305.

## Results

### HepaRG cells express hepatocyte markers after one week of differentiation

**Figure 1:**
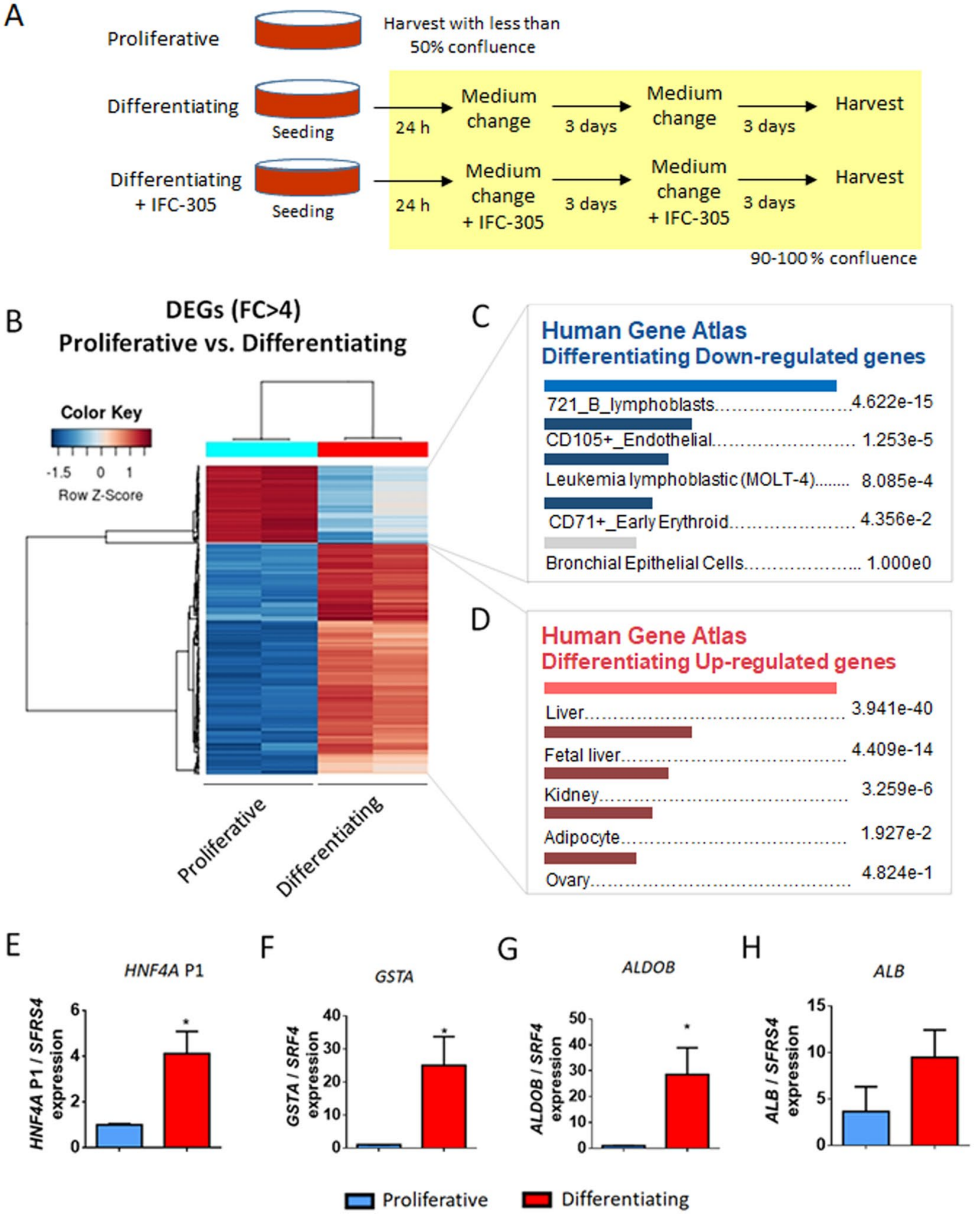
Liver transcription program is expressed in HepaRG cells at one week of differentiation. (**A**) HepaRG differentiation model. For proliferative (progenitor) condition, cells were seeded and trypsinized before reaching 50% confluence; for differentiating conditions, cells were seeded at 70–80% confluence in order to reach 100% confluence 24 h after seeding. (**B**) Transcriptome was analyzed in both conditions. Heatmap represents differentially expressed genes (DEGs) with fold change greater than four. Cell/tissues types associated with genes down-regulated (**C**) and up-regulated (**D**) in differentiating cells (EnrichR), adjusted p-values are shown. (**E–H**) Expression of hepatocyte markers was validated by RT-qPCR, data represent mean ± SEM 3 independent cultures/condition; *Statistical difference (p < 0.05).

HepaRG cells are bipotent liver progenitor cells that differentiate *in vitro* after 4 weeks into either hepatocytes or cholangiocytes. Our group previously found a TET1-dependent switch from methylated to hydroxymetylated DNA status at *HNF4A* promoter P1 in HepaRG cells, triggering differentiation at one week of cell culture^26^. In order to determine the gene expression profile at this stage of hepatocyte differentiation (Fig. 1A), RNA was isolated and a transcriptome analysis was performed to identify differentially expressed genes (DEGs) (Fig. 1B). We found 4175 DEGs upon one week of differentiation. Down-regulated genes (n = 2066 probes, corresponding to 1772 hg19-annotated genes) were related to lymphoblasts and endothelial cells (Fig. 1C), and associated with *E2F4* transcriptional program (Supplementary Fig. S1E), signalling pathways involved in cell cycle progression, biological process related with DNA metabolism and replication, and molecular functions implicated in DNA dependent ATPase activity (Supplementary Fig. S1F–H). In contrast, over-expressed genes (2109 probes, corresponding to 1822 hg19-annotated genes) were highly associated with liver and foetal liver cells (Fig. 1D), and were enriched in targets of the *HNF4A* transcription program (Supplementary Fig. S1A). Pathways and ontologies related with over-expressed genes included biological oxidation and metabolism (fatty acids, regulation of lipids, and triglyceride homeostasis, oxidoreductase, and endopeptidase and alcohol dehydrogenase activities) (Supplementary Fig. S1B–D). We assessed expression levels of hepatocyte markers over-expressed in transcriptome data and validated the overexpression of *HNF4A* P1 isoforms, *GSTA*, and *ALDOB* (Fig. 1E–H; analysed regions for *HNF4A* P1 are shown in Supplementary Fig. S2).

Altogether, these results indicate that after one week of differentiation, HepaRG cells have turned on a hepatocyte-like expression program, while proliferative related genes become progressively silenced.

### Early HepaRG differentiation is associated with a global increase in 5hmC

**Figure 2:**
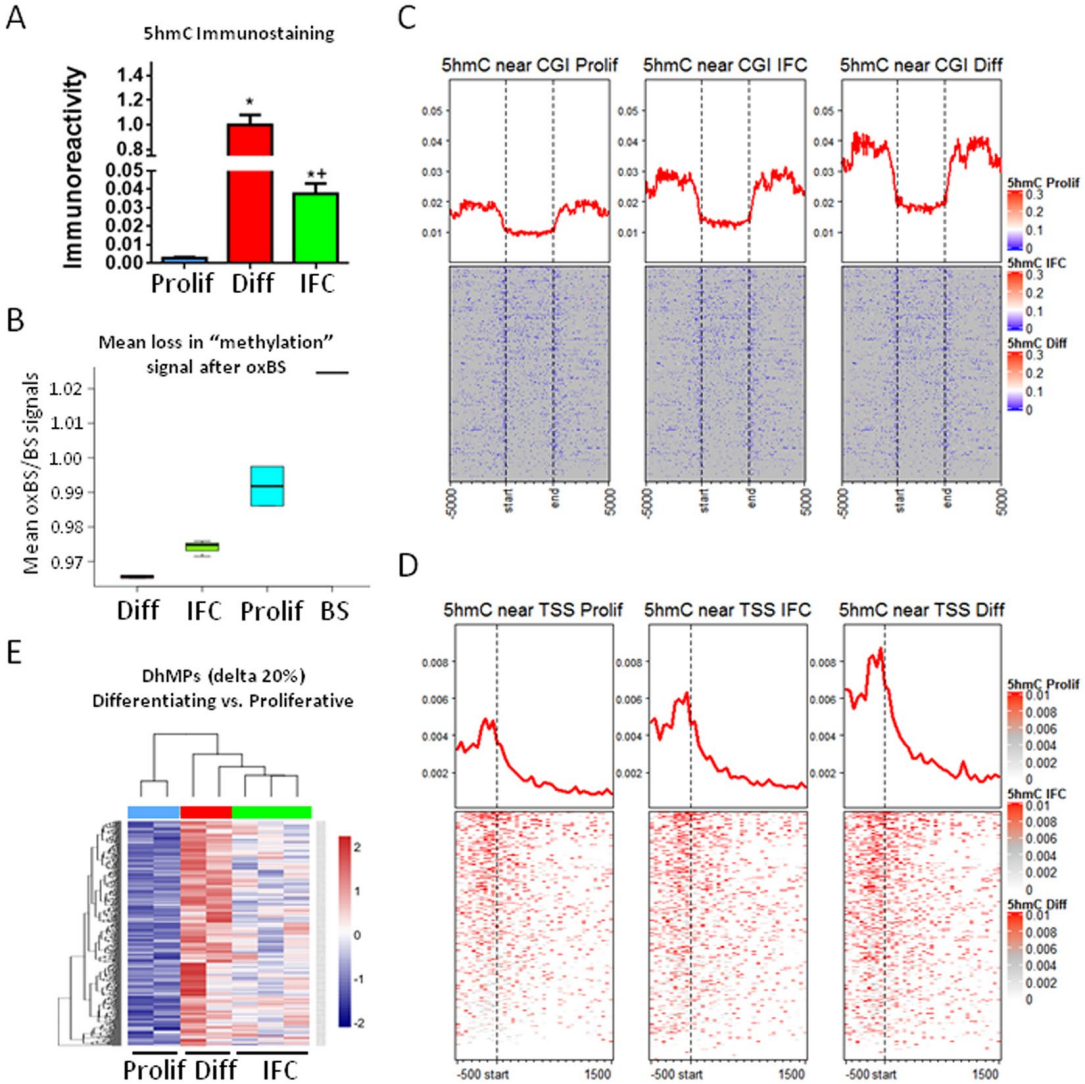
5hmC genome-wide enrichment during hepatocyte differentiation. (**A**) Immunofluorescence of 5hmC in proliferative (Prolif), differentiating (Diff) and differentiating + IFC-305 (IFC) HepaRG cells. Quantification barplots of 5hmC signal are based on mean ± SEM from 3 fields per group. *Statistical difference (p < 0.05) compared with proliferative, or *^+^(p < 0.05) compared with proliferative and differentiating cells. (**B**) The same conditions were assessed for base-resolution 5mC/5hmC, using BS and OxBS followed by EPIC beadarray hybridization (see Methods). Proportion of signal loss after oxidation (mean oxBS signal / mean BS signal) was used as an estimation of global 5hmC in each condition. BS represents the variation between two BS technical replicates. Global distribution of 5hmC for one representative sample of each condition, according to CpG islands (CGI) (**C**) and transcription start sites (TSS) (**D**). In both cases, 5hmC levels are averaged across all hg19-annotated genomic regions. (**E**) Heatmap showing hydroxymethylome comparison between differentiating and proliferative cells. Differentially hydroxymethylated positions (DhMPs) were filtered by the magnitude of change in methylation (delta beta) of at least 20% and p-adjusted value <0.05. Two independent cultures were used for proliferative and differentiating cells, and three independent cultures for differentiating + 1 mM IFC-305.

Considering that at one week of HepaRG differentiation there is TET1-mediated 5hmC enrichment on *HNF4A* promoter P1^26^ and the transcriptome already reflects a hepatocyte-like profile, we chose this time point to assess 5hmC levels of the HepaRG cell line compared to its proliferative state. Immunostaining against 5hmC reveals the presence of this modified cytosine in differentiating cells, in contrast with its almost complete absence in proliferative HepaRG cells (Fig. 2A and Supplementary Fig. S3).

Next, we assessed the hydroxymethylome at base resolution using oxidative bisulfite, which allows the identification of 5mC through the oxidation of 5hmC to 5-formylcytosine (5fC) with KO_4_Ru^35^. To this end, we isolated DNA from proliferative and differentiating HepaRG cells and performed oxidative and conventional bisulfite conversion (oxBS and BS, respectively), followed by hybridization on Infinium EPIC arrays. Using only oxBS signal (which corresponds to the “real” 5mC content), we observed the expected distribution of 5mC in CpG islands (CGIs) and transcription start sites (TSS) (Supplementary Fig. S4). Although no global 5mC differences were evident between conditions, we identified 3351 differential methylated positions (DMPs) displaying lower methylation after one week of differentiation (Supplementary Fig. S5A).

Roughly, a loss of signal after oxBS relative to conventional BS indicates the presence of 5hmC. While analysis of genomic data showed almost no signal loss between oxBS and BS in proliferative cells, a significant global loss was evident in differentiating cells suggesting a global gain in 5hmC during the first week of differentiation (Fig. 2B). Such gain in 5hmC was consistent, regardless of relative location across CGIs and TSS (Fig. 2C,D).

Next, we studied 5hmC by directly comparing oxBS and BS data. While no significant differences were observed in proliferative cells, we identified a gain of 5hmC at 11766 sites in differentiating cells, defined by a significant reduction of methylation signal after oxBS of at least 10% (5hmC “peaks”). Differential hydroxymethylation performed at these sites revealed 6952 differentially hydroxymethylated positions (DhMPs) between proliferative and differentiating cells (Fig. 2E). In addition, we identified 2482 differentially hydroxymethylated regions (DhMRs). Among these, a 3-CpGs region on *HNF4A* was identified as well as a 21-CpGs region, a 5-CpGs region and two 3-CpGs regions on *IDH3G, TET1* and *TET3* genes, respectively (these *loci* can be seen in the accompanying UCSC Genome Browser URL session^36^). All of these genes are involved themselves in establishment of 5hmC.

Together, these results show that while 5hmC is poorly present at the HepaRG progenitor stage, it is enriched at multiple genomic locations upon entering hepatocyte differentiation.

### Genomic and functional context of differentially hydroxymethylated sites associated with early hepatocyte differentiation

**Figure 3:**
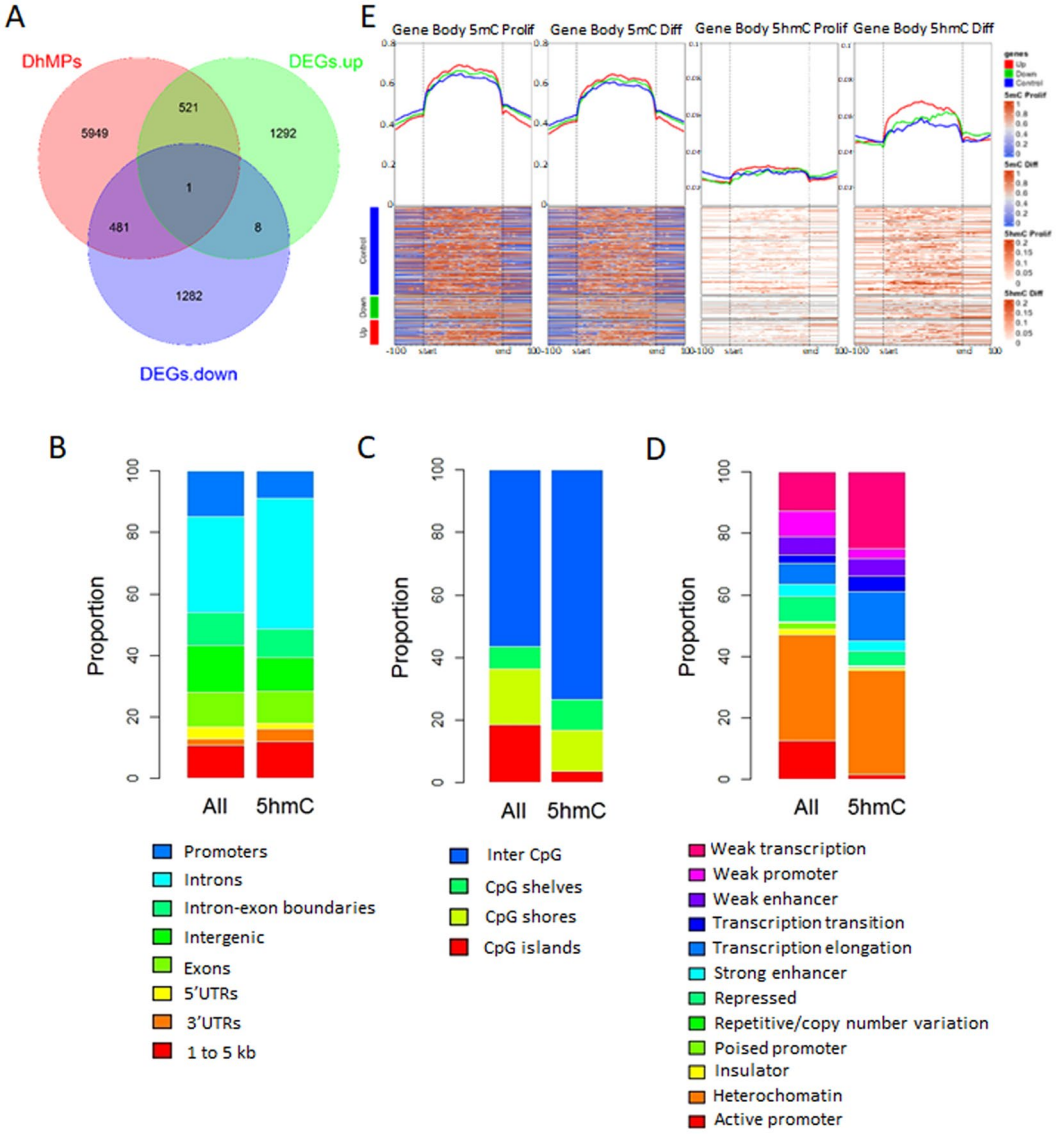
Association between 5hmC and differential expression. (**A**) Venn diagram representation of the overlaps between 5hmC differentiated positions (DhMPs) and differential expressed genes (DEGs). For this analysis, DhMPs were annotated to its nearest gene. 5hmC levels are averaged across all hg19-annotated genomic regions, in turn divided into control, up-regulated, or down-regulated genes. DhMPs were also annotated according to gene features (**B**), distribution according to CGIs (**C**), and HepG2 chromatin states (ChromHMM) (**D**). For each genomic context, distribution is shown separately for all DhMPs, and all EPIC beadarray probes, as a control. (**E**) Metagene heatmaps showing the distribution of 5mC (two left panels) and 5hmC (two right panels) in proliferative and one-week differentiated HepaRG cells. All panels show the average distribution of 5mC/5hmC values in the bodies of genes known to be up- (red) or down- (green) regulated after one week of differentiation. Housekeeping genes that did not display any significant change are shown in blue. Two independent cultures were used for proliferative and differentiating cells.

In order to know whether there is a relationship between 5hmC enrichment and gene expression, we compared the nearest associated gene of DhMPs with DEGs associated with one week of differentiation (described above). Out of 6952 DhMP-associated genes, 522 and 482 fall near an up- or a down-regulated DEG, respectively (Fig. 3A). DhMPs associated with increased gene expression were related with liver and foetal liver cell types, and enriched in pathways involved in androgen receptor (*AR)* and Nuclear Factor Erythroid 2-Related Factor 2 (*NFE2L2*) transcription programs as well as part of metabolism signalling pathways (Supplementary Fig. S6A–C). DhMPs associated with down-regulated genes, were related with B lymphoblasts and enriched in pathways involved in cell cycle control, such as the *E2F4* transcription program (Supplementary Fig. S6D–F).

In order to explore a less evident role of 5hmC enrichment on gene expression, we compared the distribution of DhMPs relative to different genomic annotations. DhMPs were enriched in intronic regions, and depleted from promoters and CGIs (Fig. 3B,C). In addition, when we assessed genomic annotated features of liver HepG2 cells, we found an over-representation of DhMPs in weak transcription and transcription elongation loci (Fig. 3D). Next, we mapped 5mC and 5hmC at gene bodies in proliferative and one-week differentiating HepaRG cells. Interestingly, 5mC content was higher in upregulated genes even at the proliferative level, with an overall reduction after triggering differentiation (Fig. 3E). Moreover, we found an overall increase in 5hmC. Despite the lower levels of 5hmC, the increase in 5hmC is more evident in the subset of upregulated genes after one-week of differentiation (Fig. 3E). When we focused on gene bodies, rather than the entire genes, there was a significant increase of 5hmC from Proliferative (2.2%) to one week Differentiating cells (4.1%); p-value < 0.01; and from one week Differentiating cells to IFC (3.5%); p-value < 0.01. Notably, the aforementioned increase was significantly higher at gene bodies with upregulated gene expression (p-value = 0.01252) compared to downregulation.

Besides the significant increase of 5hmC, we also detected a low magnitude reduction of 5mC at the corresponding gene bodies as follows: from Proliferative (51.7%) to one week Differentiating cells (49.1%); p-value < 0.01, and from one week Differentiating cells to IFC (50.5%); p-value < 0.01.

In summary, these results show that only 14% of 5hmC enrichment during HepaRG differentiation can be directly associated with gene expression changes at the neighbouring genomic location. However, 5hmC content is overall higher at the gene bodies of upregulated genes, in line with other base-resolution analyses of the association between gene expression and 5hmC [reviewed in^37^].

### Adenosine derivative exposure and TET inhibition impair hepatocyte-like differentiation

Considering the genome-wide increase in 5hmC upon early HepaRG differentiation, we evaluated if a metabolic stimulation of *trans-*methylation reactions could impact on 5hmC levels, triggering a modification of the hepatocyte differentiation process. To assess this question, we used a previously described adenosine derivative, IFC-305, which is able to regulate metabolic fluxes, favouring SAM availability and modulating DNA methylation dynamics *in vivo*^28^.

**Figure 4:**
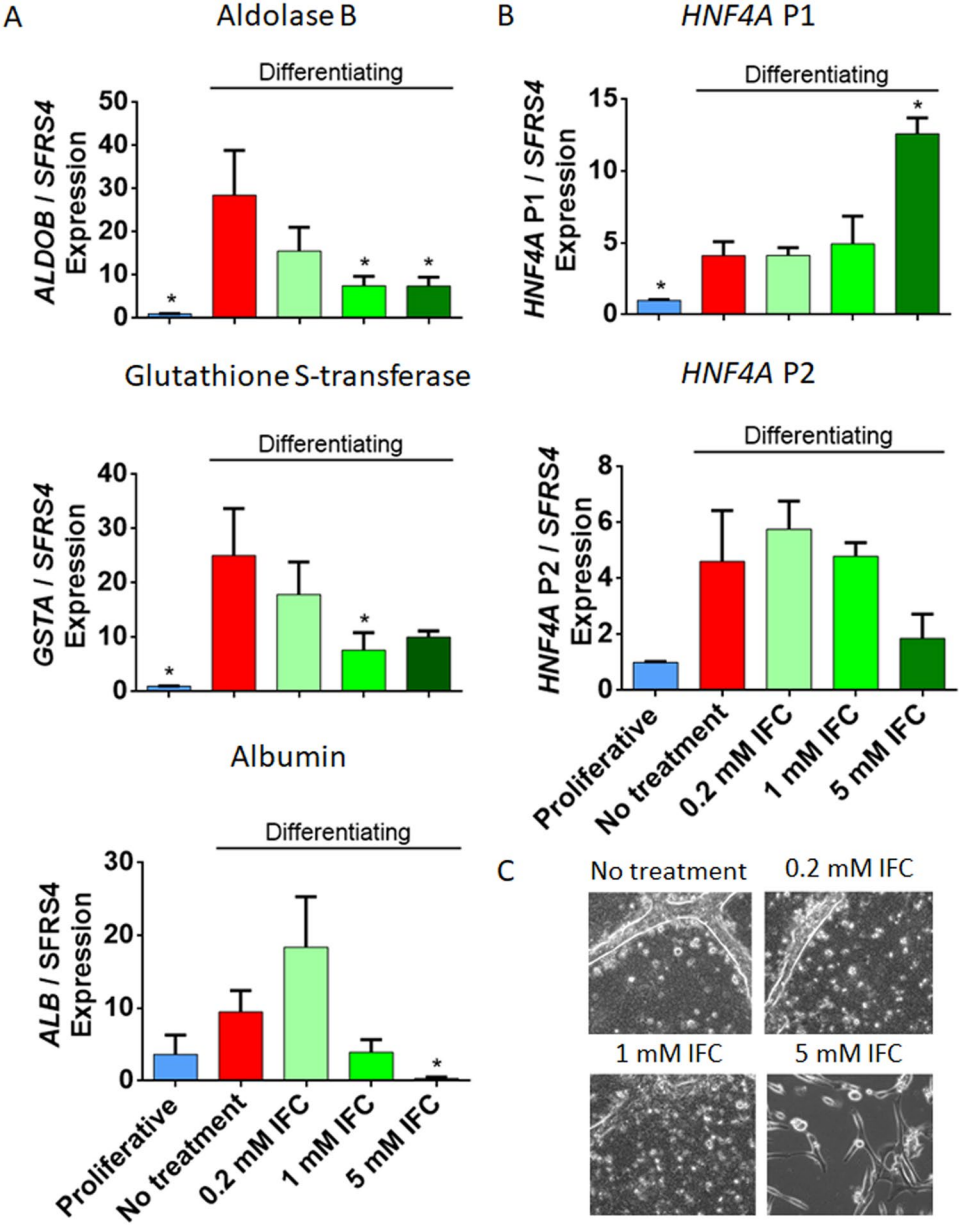
Impaired hepatocyte marker expression upon adenosine derivative exposure. (**A**) Expression of hepatocyte markers was assessed by RT-qPCR, data represent mean ± SEM 3 independent cultures/condition. (**B**) Expression of isoforms from *HNF4A* promoters 1 and 2 was assessed by RT-qPCR, data represent mean ± SEM 3 independent cultures/condition. (**C**) Phase contrast images showing HepaRG differentiated phenotype after 20 days of exposure to an increasing gradient of IFC-305. Representative 20x magnification images from seven independent cultures are shown. *Statistical difference (p < 0.05) when compared with differentiating non-treated cells.

Firstly, we evaluated HepaRG cell viability in response to IFC-305 exposure and we found that viability was not affected at concentrations of up to 1 mM for one week of differentiation (Supplementary Fig. S7). Next, we analysed the expression of hepatocyte markers in response to different concentrations of IFC-305 and noticed that 1 mM triggered lower values of *ALDOB* and *GSTA* in comparison with non-treated differentiating cells (Fig. 4A). Albumin expression did not change, neither on differentiating non-treated cells nor after IFC-305 treatment, except for a reduction in expression levels with 5 mM IFC-305 (Fig. 4A) compared with proliferative cells. Regarding *HNF4A* isoforms regulated by P1 gene promoter, its expression showed the same increment with 0.2 and 1 mM IFC-305 and non-treated differentiating cells, while a concentration of 5 mM induced an over-expression of these isoforms compared with differentiating and proliferative cells (Fig. 4B). On the other hand, *HNF4A* isoforms regulated by P2 gene promoter increased its expression with 0.2 and 1 mM IFC-305 on differentiating cells in comparison with proliferative cells (Fig. 4B). At the protein level, 1 mM IFC-305 was associated with a reduced HNF4α nuclear immunoreactivity signal in differentiating cells compared with non-treated differentiating ones (Supplementary Fig. S8).

This pattern of expression of hepatocyte markers suggests that HepaRG exposure to IFC-305 impairs hepatocyte differentiation. Exploring this possibility, we extended the differentiation model to a longer period (20 days). At 13 days, differentiating non treated cells show hepatocyte-like cell morphology, emergence of small polygonal cells with increased granularity and organized in well-delineated trabeculae, separated by bright canaliculi-like structures (Supplementary Fig. S9). IFC-305 delays this phenotype in a concentration-dependent manner, with 1 mM IFC-305 treated cells showing a phenotypic delay of at least one week, and 5 mM IFC-305 retaining a proliferative-like phenotype up to day 20 of differentiation (Fig. 4C and Supplementary Fig. S9). Such phenotypic variation, reduced levels of hepatocyte markers (e.g. *HNF4A* isoforms regulated by P1 gene promoter, *ALDOB* and *GSTA)*, an increased expression of *HNF4A* isoforms regulated by P2 gene promoter, and a trend of *TET1* expression reduction (Supplementary Fig. S10A), suggest that IFC-305 is able to delay hepatocyte differentiation.

With the interest to know if differentiation is regulated by acquisition of 5hmC at one week of HepaRG cell differentiation, we evaluated effects of chemical and transcriptional TETs inhibitors at concentrations that do not affect cell viability. HepaRG cells were exposed to dimethyloxalylglycine (DMOG), a cell-penetrating derivative of N-Oxalylglycine (NOG) that inhibits Fe^2+^/2-OG dependent dioxygenases such as TET enzymes^38^ or transfected with a pool siRNA against *TET1* and *TET2*. 200 μM DMOG is able to reduce *TET1* expression when compared to non-treated cells (Supplementary Fig. S10A). A similar behavior is observed for siTETs, as expected. Therefore, both chemical and transcriptional TETs inhibitors reduce expression of *TET1* at one week of HepaRG differentiation. In both conditions (200 μM DMOG and siTETs) the expression of the master regulator of hepatocyte differentiation *HNF4A* P1 is reduced, compared to non-treated cells and siCTRL respectively (Supplementary Fig. S10C). This indicates that specific inhibition of *TET1* reduced the expected levels of *HNF4A* P1 expression at one week of differentiation. Previously we demonstrated that this reduction of *HNF4A* P1 correlated with 5hmC absence on P1 promoter^26^. Together these results suggest that interference with *TET*s expression levels alters the regulation of hepatocyte transcription program from the *HNF4A locus*.

Analysis of hepatocyte expression markers indicates that 200 μM DMOG exhibits similar behavior to IFC-305: it strongly reduces the expression of *ALDOB, GSTA*, and *ALB*. Meanwhile, siTETs increased the expression of *ALDOB*, showed a similar trend in *GSTA*, and did not induce changes in *ALB* expression levels (Supplementary Fig. S10E–G). No phenotypic effect was observed with neither of the two treatments (DMOG and siTETs) at one week of HepaRG differentiation (Supplementary Fig. S10H–J). Therefore, both TET inhibitors affect in different way hepatocyte expression markers at one week of HepaRG cell differentiation.

Despite the limitation of lacking specificity against TETs, we chose the nucleoside analogue IFC-305 for further experiments, as it represents a way to counteract the methyl-donor depletion that may occur in several pathological conditions as cirrhosis^28^ and HCC^39^, properties that support it as a non-toxic compound for the liver.

### Disruption of the metabolic environment by adenosine derivative modifies the 5hmC landscape during hepatocyte-like differentiation

**Figure 5:**
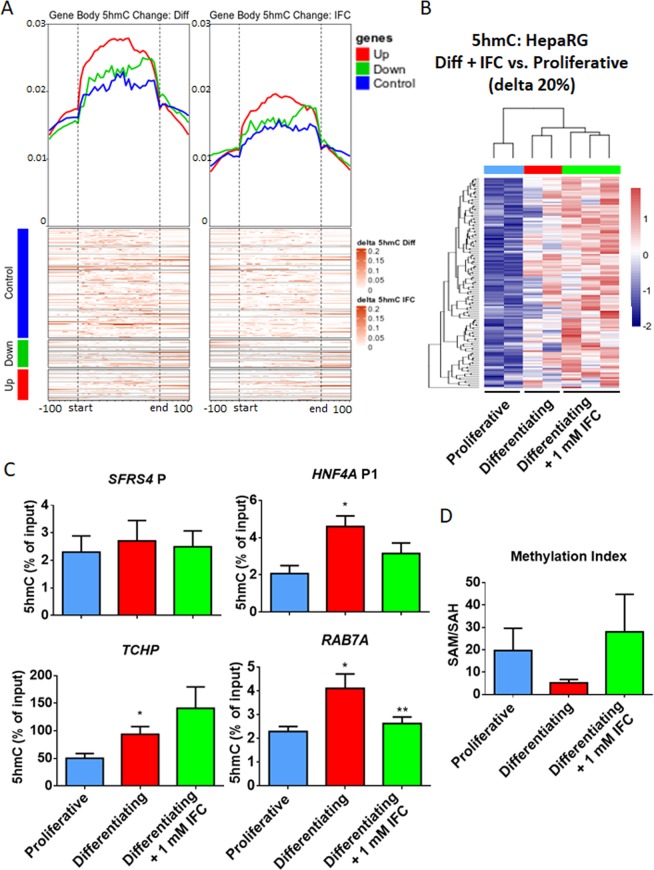
Reduced 5hmC enrichment under methyl-donor perturbation. 5hmC in proliferative, differentiating and differentiating + IFC-305 HepaRG cells, as assessed using BS and OxBS followed by EPIC beadarray hybridization (see methods and Fig. 2). (**A**) Metagene heatmaps showing the shift of 5hmC content upon one week of HepaRG differentiation in control conditions (left panel) or after exposure to the adenosine derivative IFC-305 (right panel). The increase in 5hmC content is plotted separately for the bodies of genes known to be up- (red) or down- (green) regulated after one week of differentiation. Housekeeping genes that did not display any significant change are shown in blue. (**B**) Heatmap showing hydroxymethylome comparison between differentiating + IFC-305 and proliferative cells. Differentially hydroxymethylated positions (DhMPs) were filtered by the magnitude of change in methylation (delta beta) of at least 20% and p-adjusted value < 0.05. Two independent cultures were used for proliferative and differentiating cells, and three independent cultures for differentiating + 1 mM IFC-305. (**C**) Validation of 5hmC changes. 5hmC enrichment was assessed by hMeDIP and qPCR; *SFRS4* gene promoter was used as non-5hmC enriched control region, *HNF4A* promoter P1 was used as one week differentiation 5hmC enrichment positive control, data represent mean ± SEM 3 independent cultures/condition; *Statistical difference (p < 0.05) compared with proliferative cells. **Statistical difference (p < 0.05) compared with differentiating cells. (**D**) Content of S-adenosylhomocysteine (SAH) and S-adenosylmethionine (SAM) was assessed using HPLC in different experimental conditions and samples added with standards. Barplots indicate the methylation index, calculated as the SAM/SAH ratio, mean ± SEM from 4 cultures/condition.

Proliferative HepaRG cells were exposed to 1 mM IFC-305 in differentiating conditions during one week. Under these conditions, 5mC reduction and 5hmC accumulation are impaired, as assessed by IF and global oxBS and BS analyses (Fig. 2A,B, Supplementary Fig. S5B). Similarly, only a fraction of differentiation-related DhMPs are detected in presence of IFC-305 using base-resolution methylation bead arrays (Fig. 2E). As described above, there is a shift in 5hmC during HepaRG differentiation, particularly evident on the bodies of upregulated genes (Fig. 5A, left panel). In contrast, cells grown under IFC-305 exposure displayed a milder 5hmC accumulation (Fig. 5A, right panel). Despite this attenuated phenotype, differential hydroxymethylation was identified at 8460 gene associated CpGs sites (Fig. 5B), and 1890 regions, relative to proliferative cells. Comparing these genes with up-regulated DEGs in differentiating cells, we found an overlap of 173 genes which are associated with liver expression, according to the ARCHS4 Tissues data base (Supplementary Fig. S11).

In order to validate the increase in 5hmC through the differentiation process, we performed hydroxymethylated DNA immunoprecipitation assays (hMeDIP) on selected regions with the highest 5hmC increase. We assessed two different regions in the vicinity of *TCHP* and *RAB7A*. Furthermore, we evaluated the region where a switch in 5mC/5hmC on *HNF4A* P1 occurs, using *SFRS4* promoter as a control region (Supplementary Fig. S2). We could observe a constant level of 5hmC on *SFRS4* promoter in proliferative and differentiating cells (Fig. 5C). *HNF4A* P1 showed an increase in 5hmC content at one week of differentiation, as previously shown^26^, and such increase was affected by IFC-305 exposure. A similar behaviour was found in *TCHP* and *RAB7A loci* (Fig. 5C).

Next we asked how an adenosine derivative is able to disturb 5hmC levels triggering a differentiation delay on hepatocytes. To address this question we evaluated several components of the methionine cycle, which is the metabolic pathway responsible for biological *trans*-methylation reactions^40^. Such reactions, including DNA methylation, could be modified by SAM availability as well as SAH levels^41,42^. We previously showed that adenosine as well as IFC-305 are able to modulate SAM levels, favouring phospholipid methylation^6^ and restoring global DNA methylation and 5hmC levels in a CCl_4_-mediated cirrhosis model^28^. With this rationale, we evaluated the content of adenosine, SAM and SAH, using HPLC (Supplementary Fig. S12). A trend to reduced SAM levels was observed in differentiating cells compared with proliferative (Supplementary Fig. S12A,B,E), which was consistent with global 5mC reduction at one week of HepaRG differentiation (Supplementary Fig. S5A), with a non-significant trend to increase in SAH levels (Supplementary Fig. S12A,B,F). Methylation index (SAM/SAH ratio) derived from these data, was lower and less variable in differentiating cells (Fig. 5D). Higher and dispersed methylation index was observed in proliferative and IFC-305 exposed cells, this last correlates with an IFC-305-mediated significant increase in SAM level (Supplementary Fig. 12E), as well as with high 5mC abundance genome wide (Supplementary Fig. S5A). Adenosine content was higher in differentiating compared to proliferative cells, with the highest levels observed in differentiating + 1 mM IFC-305 cells (Supplementary Fig. S12A–C,G; standard chromatogram is shown in Supplementary Fig. S12D) which is understandable, since IFC-305 is an adenosine derivative.

These results suggest that IFC-305 generates a methylation environment which competes with the demethylation wave observed during hepatocyte differentiation, with the consequent reduction in 5hmC enrichment.

In order to determine if adenosine derivative effects could be attributed to increased SAM levels, we exposed HepaRG cells during one week of differentiation to 1 mM SAM. A reduction in cell number was observed over time (Supplementary Fig. S14A) mainly on the culture periphery. There was an over expression of *HNF4A* P1 and P2, but hepatocyte markers *ALDOB, GSTA* and *ALB* were downregulated compared with differentiating non-treated cells (Supplementary Fig. S14C). DNA methylation pathway components were also affected, being DNA methyltransferases (*DNMT1*, *DNMT3A*, *DNMT3B*) downregulated as well as transcripts corresponding to methionine adenosyltransferases enzymes (*MAT1A* and *MAT2A*) (Supplementary Fig. S14D). In terms of methylcytosine oxidation pathway components, we found an induction of the *TET1* transcript (with the same trend for *TET2*), and a reduction of isocitrate dehydrogenase enzymes transcripts (*IDH1* and *IDH2*). IDH enzymes generate α-ketoglutarate, a metabolite required for TET function and potentially linked to the impaired 5hmC enrichment observed upon SAM exposure (Supplementary Figs. S13 and S14E). Regarding IFC-305 effects, we could observe an overexpression of DNA methyltrasferases, same level of *MAT2A* transcript, we validated the subexpression of *TET1* and *TET2*, and observed an intermediate expression level of *IDH2*, compared with differentiating non-treated cells (Supplementary Fig. S14). Taken together, these results indicate that SAM exposure partially phenocopies the effect of IFC-305, in terms of reduced 5hmC enrichment and impaired acquisition of hepatocyte markers. While 5hmC reduction by adenosine derivative could be mediated by overexpression of *DNMTs* and downregulation of *TET*s, 5hmC reduction in response to SAM could also related to *IDH* dowregulation.

Broadly, these results could indicate that the metabolic state is able to influence differentiation in HepaRG cells as well as 5hmC enrichment during this process.

## Discussion

In this work, we have assessed the link between DNA methylation dynamics and the establishment of the hepatocyte transcription program after one week of HepaRG cells differentiation. We found that at one week of differentiation these cells have already triggered a hepatocyte transcriptional program, a time when there is a reduction of DNA methylation and 5hmC emerges genome-wide. While only 14% of DhMPs were associated to changes in gene expression, they were enriched in introns and depleted from promoters and CGIs. In particular, 5hmC content was overall higher at the gene bodies of upregulated genes, in line with previous studies on 5hmC distribution [reviewed in^37^]. Finally, we found that modification of metabolic environment through an adenosine derivative can reduce 5hmC enrichment associated to differentiation. This is in turn associated with reduced expression of hepatocyte markers.

Since its rediscovery in 2009^7,8^, 5hmC emerged as an intermediate of active DNA demethylation, although increasing evidence considers this modified cytosine as the “sixth base” because of its stability and its distinct functionality. Regardless of its functional meaning, 5hmC events correlate with key differentiation steps in mammal adult progenitors cells^9^. Analysis of BS and OxBS from one week differentiating HepaRG cells as well as 5hmC immunostaining (Fig. 2A,B, Supplementary Fig. S3), showed a genome-wide increase of this modified cytosine which corresponded with our previous findings on the *HNF4A P1* gene promoter^26^. This behaviour of 5hmC enrichment has been described also in tissues from every germ layer (e.g. enterocytes^43,44^, myocytes^45^, adipocytes^46,47^ and neurons^48,49^), suggesting a general process^12^.

Cell specification is accompanied by a stark transition in epigenetic landscape from a uniquely accessible state in pluripotency, to increasingly restrictive configurations in differentiated stages^50^. However, 5hmC is often related to highly expressed cell-specific genes^12,15^. This is in line with our findings of 5hmC enrichment in hepatocyte-specific transcriptional processes such as metabolic pathways, and with liver specialized functions such as synthesis of complement components, regulation of lipid metabolism, platelet degranulation and oxidoreductase activity. This suggests that 5hmC may operate in concert with mechanisms that generate a restrictive chromatin in non-hepatocyte related *loci*. Furthermore, we found DhMRs associated with *TET*s, and *IDH3G* which could be related with a 5hmC feedback loop to reinforce differentiation. In line with these findings, it has been recently described that *TET1* is regulated through DNA methylation on its own promoter^51^.

Further studies are required to assess the stability of 5hmC during hepatocyte differentiation. Of note, using oxidative bisulfite followed by quantitative methyl-specific PCR (OxBS-qMSP), we confirmed that 5hmC accumulation at the *HNF4A* P1 *locus* is transient in HepaRG cell differentiation (Supplementary Fig. S15). In a similar way, 5hmC is almost absent in that locus in terminally differentiated primary human hepatocytes (Supplementary Fig. S15). Whether a similar kinetics occurs at genome-wide level will be a subject of future analyses.

We found 5hmC enrichment in gene bodies (introns) upon differentiation, features that partially agree with a report in adult mouse liver which indicates that 5hmC is enriched in intragenic regions^12^. However, an earlier study on hepatocyte differentiation^11^ provides the closest *in vivo* model to contrast our results. In their work, they found that global 5hmC patterns were sufficient to stratify mice livers according to their age, highlighting 5hmC as an identifier of liver tissue and cell state. When we compare our list of genes differentially hydroxymethylated in HepaRG cells with those differentially hydroxymethylated during liver maturation from Thomson’s publication, we find a significant overlap of 122 genes (40% more than expected by chance using a hypergeometric test, p < 4.8e-05). Similarly, in adult healthy liver, a specific 5hmC landscape has been described, which includes a bimodal 5hmC accumulation around TSS and an increased level along gene bodies, with another peak right after transcription termination^15^. Interestingly, we noticed that the *TET3* locus might be related with distal regulatory elements, which co-localizes with an enhancer for adult liver and overlaps with H3K4me1 and H3K27ac histone mark signals in HepG2 cells, as reported on the Epigenome Road Map Consortium and ENCODE, respectively (this *loci* can be seen in the accompanying UCSC Genome Browser URL session^36^). Despite this correlation, we did not find a global overlap between intragenic 5hmC enrichment and histone marks related with enhancers (Supplementary Fig. S16).

Our finding of 5hmC enrichment during differentiation cannot be attributed to any particular cell subpopulation. It will be interesting for further projects to analyze from a single cell approach if enrichment of 5hmC at one week of HepaRG cell line differentiation correlates with expression of hepatocytes markers. In addition, a longtime problem in the field is the lack of causal evidence for a direct link between DNA methylation and gene expression. New tools of epigenetic editing may be able to provide such type of evidence, which should be the subject of future research.

In order to understand 5hmC dynamics in response to a perturbation of metabolism, we assessed the effect of an adenosine derivative (e.g. IFC-305) able to modulate SAM, 5mC and 5hmC levels during experimental cirrhosis^28^. We found that the hydroxymethylome of HepaRG cells exposed to IFC-305 presents an intermediate state between proliferative and differentiating cells (Figs. 4 and 5, Supplementary Fig. S9). This could be partially explained by the capacity of IFC-305 to increase SAM levels (Supplementary Fig. S12E). Moreover, IFC-305 influences the methylation environment through overexpression of *DNMTs* (Supplementary Fig. S14D), which favors DNA methylation. It was previously shown that IFC-305 is able to stimulate proliferating cell nuclear antigen (PCNA)^31^, which together with a complex machinery, guides DNMT1 to hemi-methylated regions for regenerating 5mC on complementary DNA strand during replication^52–57^. As such, maintaining DNA methylation through increasing DNMT1-guiding proteins, as well as stimulating cell cycle components such as CDK4, CDK6 and cyclin D1^31^, could be an alternative IFC-305 mechanism of action which explains the retention of a proliferating phenotype. On the other hand, the adenosine derivative allows a moderate expression of *TETs* (Supplementary Figs. S10A and S14E), as well as *IDH2* expression (Supplementary Fig. S14E) and enzyme activity^58^. Although 5hmC level is attenuated (Fig. 2, Supplementary Figs. S3 and S13), the modified oxidative reactions are still sufficient to allow cells to enter differentiation.

Previously, it has been shown that SAM is a key metabolite that regulates hepatocyte growth, death, and differentiation depending on its concentration. In addition, it is highly unstable with a short half-life and converts to methylthioadenosine rapidly *in vitro*^59^, molecule that mimics SAM effects but inhibits methylation and polyamine synthesis^5,59^. Due to its dual effect regulating growth and death, hepatic SAM levels need to be tightly regulated^59^. It is likely that at least part of the effect of IFC-305 exposure, including the reduced expression of hepatocyte markers, is dependent on its capacity to act as a stimulator of physiological SAM availability. In addition, we observed low expression of *DNMTs* due to SAM exposure (Supplementary Fig. S14D), in agreement with a report showning that *in vitro* SAM exposure triggers *DNMTs* downregulation^60^. From a chemical point of view it is understandable that expression of enzymes required to synthetize SAM (*MAT1A* and *MAT2A*) is decreased (Supplementary Fig. S14D) whenever there is an excess of the product they generate (e.g. SAM). On the other hand, despite a SAM-upregulation of TETs enzymes (Supplementary Fig. S14E), the lack of *IDH*s expression (Supplementary Fig. 14E) prevents the enrichment of 5hmC (Supplementary Fig. S13). Contrary to our approach where cells are exposed to a SAM-stimulating compound, a mouse model of methionine-choline-deficient diet which reduces SAM availability, did not cause a significant reduction of 5mC levels, instead inducing an up-regulation of Tet2, Tet3, thymine DNA glycosylase and apurinic/apyrimidic-endonuclease 1, and increased expression of Dnmt1 and Dnmt3a^61^. Thus, while methylation dynamics could overcome SAM deficiency, SAM increase leads to reduced 5hmC enrichment associated with a delay in differentiation (Supplementary Fig. S9).

The possibility of modulating differentiation through regulation of 5hmC represents a relevant observation. Indeed, some studies indicate that one of the early alterations in HCC is a significant decrease of global genomic 5hmC, a behaviour also observed in hepatitis B virus infection^17^, cirrhosis^28^, and during hepatic stellate cells *trans*-differentiation to myofibroblasts^62^. Altered patterns of 5hmC are not only detectable in tumour-normal tissue pairs from resections or biopsies, but also in circulating DNA from cancer patients^37^, positioning 5hmC as a potential biomarker for early cancer detection. Moreover, the possibility to modify cell identity through regulation of 5hmC distribution and levels represents an innovative pharmacological method to prevent or reverse certain pathologies.

## Conclusions

Our findings illustrate how early differentiation of liver progenitor cells involves a genome-wide increase in 5hmC. This 5hmC increase, more evident in the body of over-expressed genes, matches the activation of a liver transcriptional program at one week of differentiation. Moreover, we found that the metabolic state is an important regulator of differentiation in this context, and modification of this environment with an adenosine derivative impairs the acquisition of hepatocyte markers as well as the genome-wide enrichment of 5hmC. These results illustrate the interplay between metabolic environment and chromatin regulation on cell differentiation, linking 5hmC to the acquisition of cell identity, and opening the door to this modified cytosine as a potential biomarker for early detection of liver pathologies related with aberrant de/differentiation such as liver cancer.

## Methods

### Chemicals

Reagents were from Sigma Chemical Co. (St. Louis, MO). IFC-305 is the aspartate salt of adenosine: 2-aminosuccinic acid-2-(6-amino-9*H*-purin-9-yl)-5-(hydroxymethyl) tetrahydrofuran-3,4-diol (1:1). It was synthesized in the laboratory in agreement with UNAM patent 207422.

### HepaRG cell culture

Human HepaRG cells (Biopredic) were cultured as follows. Differentiating cells (4.4 × 10^4^/cm^2^) were grown for one week in William’s E medium (Gibco 12551-032) supplemented with 10% fetal bovine serum (Eurobio CVFSVF0001), 1x penicillin/streptomycin (Gibco 10378016), 5 μg/mL insulin (Sigma I9278) and 3 × 10^−5^ mM hydrocortisone (Sigma H0888) in 6 well plates (Fig. 1), medium was replaced 24 h after seeding and once more after 3 days. For proliferative conditions, cells were seeded at low confluence, avoiding cell to cell contact to prevent cell differentiation. Treatment with IFC-305 began 24 h after cell seeding, and cells were re-treated at day 3 of culture when medium was replaced (Fig. 1). Cells exposed to Dimethyloxaloylglysine (DMOG) (Biotechne CAS# 89464-63-1) followed the same treatment scheme than IFC-305, with corresponding concentrations. Cells exposed to S-adenosylmethionine (SAM) in its p-toluene sulfonate salt form^63–66^ (Sigma A-2408) were treated eveery 24 h with 1 mM concentration in fresh medium which is equivalent to the concentration used for IFC-305. Treatment began 24 h after cell seeding until day 6. The three compounds were directly solubilised in William’s E medium. For gene silencing, pool siRNAs against *TET1* and *TET2* or siRNA CTRL (Dharmacon, On-Target Plus siRNA) were transfected on HepaRG cells (4.4 × 10^4^/cm^2^), seeded 24 h earlier on 12-wells plates, as previously described^26^. Cells were washed and medium was replaced 12 h after transfection, and at each time of medium changing, according to the scheme of Fig. 1. Cells were harvested 7 days after seeding.

### Transcriptome analysis

RNA was isolated, directly from culture plate, with TRIzol (Invitrogen 15596018) and treated with DNase (Invitrogen 18068-015). Using the Illumina TotalPrep-96 RNA Amp Kit (Ambion 4393543), 500 ng of RNA was reverse transcribed into cDNA, which undergoes second strand synthesis to become a template for T7 RNA polymerase and thereby labelled with biotin-UTP. Labelled cDNA (2000 ng) was hybridized to the Illumina HumanHT-12 v3 (Illumina) and processed according to the manufacturer’s protocol. Slides were scanned immediately using Illumina BeadStation iScan (Illumina). Assays were performed in two independent cell cultures/condition.

### Quantitative RT-PCR

cDNA synthesis was performed from 2 μg of total RNA using M-MLV Reverse Transcriptase (Invitrogen 28025-013) and random primers. All quantitative PCR assays were performed independently in 3 cell cultures/condition in duplicate, using Mesa green qPCR 2x MasterMix Plus (Eurogentec 05-SY2X-06 + WOU) on a CFX96 PCR system (Bio-Rad). Primers were described previously^26^ and indicated in Supplementary Table S1. Relative expression was calculated according ΔΔCt method as follows: Expression level = 2^−(ΔΔ Ct), where ΔCt = (average Ct _evaluated gene_ - average Ct _housekeeping gene_), and ΔΔCt = ΔCt - Average _ΔCt control condition_). Normalization was performed against Proliferative cells, using *SFRS4* as housekeeping gene.

### Cell survival evaluation

Differentiating and IFC-305 treated HepaRG cells cultured for one week were trypsinized and counted using TC20 automated cell counter (Bio-Rad) with dual chamber slides and trypan blue (Bio-Rad 1450003). Total cell and live cell numbers were determined automatically and % survival was determined by comparing live cells in each condition, with the mean of non-treated live cells considered as 100%.

### Immunofluorescence

HepaRG cells (400000 /well) were seeded in 6-wells plates with 4 coverslips/well, and treated according to differentiation model with or without IFC-305. 100000 cells were seeded in the same conditions during 24 h and were designated as proliferative cells. At different time points cells were washed with PBS, fixed in 4% formaldehyde and washed twice with PBS. Primary antibodies for immunofluorescence were anti-HNF4α (Cell signalling 3113 S) diluted 1:200, and anti-5hmC (Active Motif 39769) diluted 1:400. Coverslips were incubated overnight at 4 °C. Then, coverslips were incubated 1 h at room temperature with 1:200 Alexa Fluor 488 goat anti-rabbit IgG (Invitrogen) and 1:200 Alexa 568 goat anti-rabbit IgG (Invitrogen) respectively. After incubation, coverslips were washed and mounted on a slide with a mounting medium containing DAPI (Vectashield). Fluorescence was visualized with an Olympus Inverted Microscope model IX71 and images captured with Evolution/QImaging Digital Camera, through Image Pro Plus software (Media Cybernetics). Negative controls were performed without primary antibodies. Settings for image acquisition were: gain 8, gamma 1, offset −1 000; white balance R 1.240, G 1.000, B 1.40; for DAPI (blue), exposure time was up to 50 ms; for Alexa 488 (green), exposure time was up to 800 ms; for Alexa 568 (red), exposure time was up to 4.5 s. Data analysis was performed using measure tool and integrative 3D surface plot plugin from ImageJ (National Institutes of Heatlh, https://imagej.nih.gov/).

### Immunoblotting

Equal amounts of protein lysates (30–50 μg) were separated by SDS-PAGE and electrotransferred to Immobilon-P membranes (Millipore). Primary antibodies specific to P1- and P2-driven isoforms of HNF4A (R&D Systems) have been previously described^26^.

### Oxidative bisulfite and methylation bead arrays

For genomic DNA isolation, 500 μL of lysis buffer (50 mM Tris-HCl pH 8, 100 mM EDTA, 100 mM NaCl, 1% SDS and 0.5 mg/mL proteinase K) were added to wells from 6-well plates, previously washed twice with PBS 1×. Cells were detached by pipetting and suspension was incubated 2 h at 55 °C. 200 μL of 6 M NaCl was added, sample was mixed and centrifuged 10 min at full speed in an Eppendorf 5415D Benchtop Microcentrifuge. Supernatant was recovered and DNA was precipitated with 500 μL isopropanol, washed with 500 μL 70% ethanol and dried by inverting the tube on a tissue. DNA was resuspended with 25 μL injectable water. 1000 ng DNA was oxidative bisulfite- and conventional bisulfite-converted using TrueMethyl Seq Kit (Cambridge Epigenetix) according to manufacturer’s instructions. This technique oxidises 5hmC to 5fC which is read as thymine (non-modified cytosine and 5mC are read as thymine and cytosine respectively, as in conventional bisulfite)^35^. Converted DNA was analysed with MethylationEPIC arrays (Illumina) using recommended protocols for amplification, labelling, hybridization, and scanning. For proliferative and differentiating cells, each methylation analysis was performed in two independent culture wells. For IFC-305, treatment was performed in triplicates. Oxidative bisulfite followed by quantitative methyl-specific PCR (oxBS-qMSP) was performed as described^67^, using primers specific for *HNF4A* P1 and P2 promoters (Supplementary Table S3).

### Bioinformatic analyses

Raw expression and methylation data were imported and processed using R/Bioconductor packages for Illumina bead arrays^68^. Data quality was inspected using boxplots for the distribution of expression signals, and inter-sample relationship using multidimensional scaling plots and unsupervised clustering. For methylation data, we removed low quality probes with a detection P value > 0.01 in more than 10% of the samples. Following swan (Subset-quantile Within Array) normalization^69^ implemented in the minfi package^70^, 5hmC “peaks” were identified by subtracting the signal of oxidative bisulfite from the signal of conventional bisulfite. Next, we performed Maximum Likelihood Estimate (MLE) of 5mC and 5hmC (oxBS.MLE) with the ENmix package^71^. Using a binomial model at each CpG locus in each sample, oxBS.MLE outputs a matrix of MLEs of 5mC levels and a matrix of MLEs of 5hmC levels, setting as NA any negative value. To define differentially expressed genes (DEGs), differentially methylated positions (DMPs) and differentially hydroxymethylated positions (DhMPs), we modelled experimental conditions as categorical variables in a linear regression using an empirical Bayesian approach^72^. Differentially methylated and hydroxymethylated regions (DMRs and DhMRs, respectively) were identified with the DMRcate package using the recommended proximity-based criteria^73^. Comparisons with an FDR-adjusted P value below 0.05 were considered statistically significant. In addition, only DhMPs with at least 10% change in 5hmC were considered as significant for any given comparison, as this has been suggested as a threshold for the sensitivity of this technique^74^. DEGs, DhMPs, and DhMRs were further analyzed to determine functional pathways and ontology enrichment using EnrichR^75^. Genomic context annotations, including chromatin states (ChromHMM) were performed with the ChIPseeker^76^ and Annotatr^77^ packages. EnrichedHeatmap package^78^ was used for summary heatmaps of genomic context. A list of human housekeeping genes was downloaded from^79^. All expression and methylation data have been deposited to the Gene Expression Omnibus repository (SuperSeries GSE130849, SubSeries GSE130844 and GSE130848).

### DNA immunoprecipitation

80 μL of aqueous genomic DNA (100 ng/μL) were sonicated in Covaris microTUBE AFA Fiber Pre-Slit Snap-Cap 6×16 mm with Covaris sonicator S220 to obtain fragments between 400–800 bp (Temperature < 7 °C, peak power 105.0, duty factor 5.0, Cycles/Burst 200 and time 40 s). 130 ng DNA was used per immunoprecipitation. Auto hMeDIP kit (Diagenode C02010033) was used according to manufacturer instructions. Precipitation was adjusted to 15 h for mixing at 4 °C and middle mix speed; for washes mixing time was 8 min, at 4 °C and middle mix speed. Immunoprecipitations were performed independently in 3 cell cultures/condition. 5 μL of immunoprecipitated hydroxymethyl DNA was analysed for enriched 5hmC regions according to oxBS data (regions with at least 3 CpGs containing a DhMP with 10% difference between proliferative and differentiating cells (Delta 10%)), *SFRS4* gene promoter was used as control of non-5hmC enrichment. All quantitative PCR assays were performed in duplicate with Mesa green qPCR 2x MasterMix Plus (Eurogentec 05-SY2X-06 + WOU) on a CFX96 PCR system (Bio-Rad) using primers indicated in Supplementary Table S2. 5hmC enrichment was determined as % (hmeDNA-IP/ Total input) as follows: % (hmeDNA-IP/ Total input) = 2^[(Ct(10%input) - 3.32) - Ct(hmeDNA-IP)]x 100%, where 2 is the amplification efficiency; Ct (hmeDNA-IP) and Ct (10%input) are threshold values obtained from exponential phase of qPCR for the hydroxymethyl DNA sample and input sample respectively; the compensatory factor (3.32) is used to take into account the dilution 1:10 of the input.

### Adenosine, S-adenosylmethionine and S-adenosylhomocysteine quantification by HPLC

Adenosine, SAM and SAH levels were determined modifying protocols previously described^80,81^. Briefly, 10 × 10^6^ proliferating HepaRG cells were seeded in a 15 cm Petri dish; 24 h after seeding, cells were either cultured with or without IFC-305 for one week to differentiate, or collected for the Proliferative condition. Next, cells were frozen in liquid nitrogen and preserved at −70°C. Frozen cells were harvested with 1.5 mL 0.03% trifluoroacetic acid in 90% Methanol. Samples were incubated 10 min at room temperature and passed through a Dounce homogenizer. Obtained suspensions were sonicated twice for 1 min in a Bransonic 220 and centrifuged at 16 000 g for 13 min at 4 °C. Supernatants were recovered and centrifuged until dry in an Eppendorf 5301 Concentrator Centrifugal Evaporator at 45 °C. Samples were reconstituted with 200 μL MilliQ water and protein concentration was determined by Bradford assay. Samples were diluted to 1.2 μg/μL protein and 100 μL 1.6 M HClO_4_ was added to 300 μL of diluted sample (0.4 M HClO_4_ final concentration) before incubating for 10 min on ice. 10 μL of 5 M K_2_CO_3_ were added to samples and incubated for 10 min on ice. Sample dilutions were centrifuged at 14000 rpm during 5 min in an Eppendorf 5415 C Micro-Centrifuge. Recovered supernatants were filtered using Phenex PTFE 4 mm syringe filters and 95 μL supernatant + 5 μL of 400 mM adenosine and 400 mM SAM standards mix were injected to HPLC Knauer E4310 (SAM standard contains SAH contamination, which was determined using SAH standard curve (data not shown)). Samples were separated in an ACE 5 C 18 column (150 × 4.6 mm) (Advanced Chromatography Technologies LTD) using a mobile phase consisting of 8 mM sodium heptanesulfonate, 40 mM ammonium phosphate monobasic and isocratic 15% methanol pH 3, at 1 mL/min flux. Detection was measured at 254 nm absorbance, and separation total runtime of 60 min. Peaks were analyzed using EUROCHROME for windows version 3.05 (Knauer GmbH).

### Statistical analysis

R/Bioconductor packages were used for bead array analyses, as described above. Plots were generated using Graph Pad Prism 5.0 (Graph Pad Software, Inc., La Jolla CA) for Windows. Wilcoxon test were used for unpaired analyses comparing average between conditions, and it was performed on RStudio 1.1.463 (RStudio, Inc.). P Values < 0.05 were consider statistically significant.

## Supplementary information

Supplementary Data.

## Data Availability

Datasets generated during the current study have been uploaded to the GEO repository (SuperSeries GSE130849, SubSeries GSE130844 and GSE130848).

## References

[1] Recillas-Targa F (2014). Interdependency between genetic and epigenetic regulatory defects in cancer. Methods Mol. Biol..

[2] Laird A, Thomson JP, Harrison DJ, Meehan RR (2013). 5-hydroxymethylcytosine profiling as an indicator of cellular state. Epigenomics.

[3] Smith ZD, Meissner A (2013). DNA methylation: roles in mammalian development. Nature reviews. Genetics.

[4] Tehlivets O, Malanovic N, Visram M, Pavkov-Keller T, Keller W (2013). S-adenosyl-L-homocysteine hydrolase and methylation disorders: yeast as a model system. Biochim. Biophys. Acta.

[5] Mato JM, Corrales FJ, Lu SC, Avila MA (2002). S-Adenosylmethionine: a control switch that regulates liver function. FASEB J..

[6] Chagoya de Sanchez V (1991). Twenty-four-hour changes of S-adenosylmethionine, S-adenosylhomocysteine adenosine and their metabolizing enzymes in rat liver; possible physiological significance in phospholipid methylation. Int. J. Biochem..

[7] Kriaucionis S, Heintz N (2009). The nuclear DNA base 5-hydroxymethylcytosine is present in Purkinje neurons and the brain. Science.

[8] Tahiliani M (2009). Conversion of 5-methylcytosine to 5-hydroxymethylcytosine in mammalian DNA by MLL partner TET1. Science.

[9] Ecsedi S, Rodríguez-Aguilera JR, Hernández-Vargas H (2018). 5-Hydroxymethylcytosine (5hmC), or How to Identify Your Favorite. Cell. Epigenomes.

[10] Ivanov M (2016). Single base resolution analysis of 5-hydroxymethylcytosine in 188 human genes: implications for hepatic gene expression. Nucleic Acids Res.

[11] Thomson JP (2013). Dynamic changes in 5-hydroxymethylation signatures underpin early and late events in drug exposed liver. Nucleic Acids Res.

[12] Lin IH, Chen YF, Hsu MT (2017). Correlated 5-Hydroxymethylcytosine (5hmC) and Gene Expression Profiles Underpin Gene and Organ-Specific Epigenetic Regulation in Adult Mouse Brain and Liver. Plos one.

[13] Thomson JP (2016). Loss of Tet1-Associated 5-Hydroxymethylcytosine Is Concomitant with Aberrant Promoter Hypermethylation in Liver Cancer. Cancer Res..

[14] Ye C (2016). Whole-genome DNA methylation and hydroxymethylation profiling for HBV-related hepatocellular carcinoma. Int. J. Oncol..

[15] Li X, Liu Y, Salz T, Hansen KD, Feinberg A (2016). Whole-genome analysis of the methylome and hydroxymethylome in normal and malignant lung and liver. Genome Res..

[16] Song CX (2017). 5-Hydroxymethylcytosine signatures in cell-free DNA provide information about tumor types and stages. Cell Res..

[17] Liu, J. *et al*. Global DNA 5-hydroxymethylcytosine and 5-formylcytosine contents are decreased in the early stage of hepatocellular carcinoma. *Hepatology*, 10.1002/hep.30146 (2018). 10.1002/hep.30146 30070373

[18] Meehan RR, Thomson JP, Lentini A, Nestor CE, Pennings S (2018). DNA methylation as a genomic marker of exposure to chemical and environmental agents. Curr. Opin. Chem. Biol..

[19] Lyall, M. J. *et al*. Non-alcoholic fatty liver disease (NAFLD) is associated with dynamic changes in DNA hydroxymethylation. *Epigenetics*, 1-11, 10.1080/15592294.2019.1649527 (2019). 10.1080/15592294.2019.1649527 PMC6961686 31389294

[20] Ivanov M (2013). Ontogeny, distribution and potential roles of 5-hydroxymethylcytosine in human liver function. Genome biology.

[21] Cannon MV, Pilarowski G, Liu X, Serre D (2016). Extensive Epigenetic Changes Accompany Terminal Differentiation of Mouse Hepatocytes After Birth. G3 (Bethesda).

[22] Chaker D (2018). Inhibition of the RhoGTPase Cdc42 by ML141 enhances hepatocyte differentiation from human adipose-derived mesenchymal stem cells via the Wnt5a/PI3K/miR-122 pathway: impact of the age of the donor. Stem cell research & therapy.

[23] Seeliger C (2013). Decrease of global methylation improves significantly hepatic differentiation of Ad-MSCs: possible future application for urea detoxification. Cell Transplant..

[24] Lee CW (2017). DNA Methyltransferases Modulate Hepatogenic Lineage Plasticity of Mesenchymal Stromal Cells. Stem cell reports.

[25] Lewis LC (2017). Dynamics of 5-carboxylcytosine during hepatic differentiation: Potential general role for active demethylation by DNA repair in lineage specification. Epigenetics.

[26] Ancey PB (2017). TET-Catalyzed 5-Hydroxymethylation Precedes HNF4A Promoter Choice during Differentiation of Bipotent Liver Progenitors. Stem cell reports.

[27] Li W, Qin J, Wang H, Chen LB (2018). Research progress of epigenetic biomarkers in the early diagnosis and treatment of human diseases. Yi chuan = Hereditas.

[28] Rodriguez-Aguilera JR (2018). Epigenetic Effects of an Adenosine Derivative in a Wistar Rat Model of Liver Cirrhosis. J. Cell. Biochem..

[29] Rodríguez-Aguilera, J. R. *et al*. In Liver Cirrhosis - Debates and Current Challenges (ed Georgios Tsoulfas) Ch. 6, (InctechOpen, 2019).

[30] Perez-Carreon JI (2010). An adenosine derivative compound, IFC305, reverses fibrosis and alters gene expression in a pre-established CCl(4)-induced rat cirrhosis. *Int*. J. Biochem. Cell Biol..

[31] Chagoya de Sanchez V, Martinez-Perez L, Hernandez-Munoz R, Velasco-Loyden G (2012). Recovery of the Cell Cycle Inhibition in CCl(4)-Induced Cirrhosis by the Adenosine Derivative IFC-305. Int J Hepatol.

[32] Perez-Cabeza de Vaca R, Dominguez-Lopez M, Guerrero-Celis N, Rodriguez-Aguilera JR, Chagoya de Sanchez V (2018). Inflammation is regulated by the adenosine derivative molecule, IFC-305, during reversion of cirrhosis in a CCl4 rat model. International immunopharmacology.

[33] Velasco-Loyden G (2010). Prevention of *in vitro* hepatic stellate cells activation by the adenosine derivative compound IFC305. Biochem. Pharmacol..

[34] Velasco-Loyden G, Perez-Martinez L, Vidrio-Gomez S, Perez-Carreon JI (2017). & Chagoya de Sanchez, V. Cancer chemoprevention by an adenosine derivative in a model of cirrhosis-hepatocellular carcinoma induced by diethylnitrosamine in rats. Tumour Biol..

[35] Booth MJ (2013). Oxidative bisulfite sequencing of 5-methylcytosine and 5-hydroxymethylcytosine. Nature protocols.

[36] *UCSC Genome Browser, session URL*, http://genome-euro.ucsc.edu/cgi-bin/hgTracks?hgS_doOtherUser=submit&hgS_otherUserName=H%20Hernandez&hgS_otherUserSessionName=HepaRG%20oxBS (2018).

[37] Pfeifer GP, Szabo PE (2018). Gene body profiles of 5-hydroxymethylcytosine: potential origin, function and use as a cancer biomarker. Epigenomics.

[38] Rose NR, McDonough MA, King ON, Kawamura A, Schofield CJ (2011). Inhibition of 2-oxoglutarate dependent oxygenases. Chemical Society reviews.

[39] Lozano-Rosas, M. G. *et al*. Diminished S-adenosylmethionine biosynthesis and its metabolism in a model of hepatocellular carcinoma is recuperated by an adenosine derivative. *Cancer biology & therapy*, 1-14, 10.1080/15384047.2019.1665954 (2019). 10.1080/15384047.2019.1665954 PMC7012146 31552788

[40] Kharbanda KK (2007). Role of transmethylation reactions in alcoholic liver disease. World journal of gastroenterology.

[41] Garcea R (1989). Protooncogene methylation and expression in regenerating liver and preneoplastic liver nodules induced in the rat by diethylnitrosamine: effect of variations of S-adenosylmethionine:S-adenosylhomocysteine ratio. Carcinogenesis.

[42] Auta J, Zhang H, Pandey SC, Guidotti A (2017). Chronic Alcohol Exposure Differentially Alters One-Carbon Metabolism in Rat Liver and Brain. Alcohol. Clin. Exp. Res..

[43] Kim R, Sheaffer KL, Choi I, Won KJ, Kaestner KH (2016). Epigenetic regulation of intestinal stem cells by Tet1-mediated DNA hydroxymethylation. Genes Dev..

[44] Chapman CG (2015). TET-catalyzed 5-hydroxymethylcytosine regulates gene expression in differentiating colonocytes and colon cancer. Scientific reports.

[45] Zhong X (2017). Ten-Eleven Translocation-2 (Tet2) Is Involved in Myogenic Differentiation of Skeletal Myoblast Cells *in Vitro*. Scientific reports.

[46] Dubois-Chevalier J (2014). A dynamic CTCF chromatin binding landscape promotes DNA hydroxymethylation and transcriptional induction of adipocyte differentiation. Nucleic Acids Res.

[47] Yoo Y (2017). TET-mediated hydroxymethylcytosine at the Ppargamma locus is required for initiation of adipogenic differentiation. Int J Obes (Lond).

[48] Li X (2017). Ten-eleven translocation 2 interacts with forkhead box O3 and regulates adult neurogenesis. Nat Commun.

[49] Hahn MA (2013). Dynamics of 5-hydroxymethylcytosine and chromatin marks in Mammalian neurogenesis. Cell reports.

[50] Zhu J (2013). Genome-wide chromatin state transitions associated with developmental and environmental cues. Cell.

[51] Li L (2016). Epigenetic inactivation of the CpG demethylase TET1 as a DNA methylation feedback loop in human cancers. Scientific reports.

[52] Schermelleh L (2007). Dynamics of Dnmt1 interaction with the replication machinery and its role in postreplicative maintenance of DNA methylation. Nucleic Acids Res.

[53] Bostick M (2007). UHRF1 plays a role in maintaining DNA methylation in mammalian cells. Science.

[54] Harrison, J. S. *et al*. Hemi-methylated DNA regulates DNA methylation inheritance through allosteric activation of H3 ubiquitylation by UHRF1. *eLife* **5**, 10.7554/eLife.17101 (2016). 10.7554/eLife.17101 PMC5012860 27595565

[55] Qin W (2015). DNA methylation requires a DNMT1 ubiquitin interacting motif (UIM) and histone ubiquitination. Cell Res..

[56] Ishiyama S (2017). Structure of the Dnmt1 Reader Module Complexed with a Unique Two-Mono-Ubiquitin Mark on Histone H3 Reveals the Basis for DNA Methylation Maintenance. Mol. Cell.

[57] Nishiyama A (2020). Two distinct modes of DNMT1 recruitment ensure stable maintenance DNA methylation. Nat Commun.

[58] Chavez, E. *et al*. Functional, metabolic, and dynamic mitochondrial changes in the rat cirrhosis-hepatocellular carcinoma model and the protective effect of IFC-305. *J. Pharmacol. Exp. Ther*., 10.1124/jpet.116.239301 (2017). 10.1124/jpet.116.239301 28209723

[59] Mato JM, Lu SC (2007). Role of S-adenosyl-L-methionine in liver health and injury. Hepatology.

[60] Kar S (2014). Expression profiling of DNA methylation-mediated epigenetic gene-silencing factors in breast cancer. Clinical epigenetics.

[61] Takumi S (2015). The effect of a methyl-deficient diet on the global DNA methylation and the DNA methylation regulatory pathways. J. Appl. Toxicol..

[62] Page A (2016). Hepatic stellate cell transdifferentiation involves genome-wide remodeling of the DNA methylation landscape. J. Hepatol..

[63] Cabrales-Romero Mdel P (2006). S-adenosyl-methionine decreases ethanol-induced apoptosis in primary hepatocyte cultures by a c-Jun N-terminal kinase activity-independent mechanism. World journal of gastroenterology.

[64] Stiuso, P. *et al*. Protective Effect of Tyrosol and S-Adenosylmethionine against Ethanol-Induced Oxidative Stress of Hepg2 Cells Involves Sirtuin 1, P53 and Erk1/2 Signaling. *International journal of molecular sciences* **17**, 10.3390/ijms17050622 (2016). 10.3390/ijms17050622 PMC4881448 27128904

[65] Li TW (2015). S-Adenosylmethionine and methylthioadenosine inhibit beta-catenin signaling by multiple mechanisms in liver and colon cancer. Mol. Pharmacol..

[66] Tomasi ML (2012). S-adenosyl methionine regulates ubiquitin-conjugating enzyme 9 protein expression and sumoylation in murine liver and human cancers. Hepatology.

[67] Hernández-Vargas, H. & Goldsmith, C. *Quantitative analysis of methylation and hydroxymethylation using oXBS-qMSP*, https://www.protocols.io/view/quantitative-analysis-of-methylation-and-hydroxyme-52bg8an (2019).

[68] Du P, Kibbe WA, Lin S (2008). M. lumi: a pipeline for processing Illumina microarray. Bioinformatics.

[69] Maksimovic J, Gordon L, Oshlack A (2012). SWAN: Subset-quantile within array normalization for illumina infinium HumanMethylation450 BeadChips. Genome biology.

[70] Aryee MJ (2014). Minfi: a flexible and comprehensive Bioconductor package for the analysis of Infinium DNA methylation microarrays. Bioinformatics.

[71] Xu Z, Taylor JA, Leung YK, Ho SM, Niu L (2016). oxBS-MLE: an efficient method to estimate 5-methylcytosine and 5-hydroxymethylcytosine in paired bisulfite and oxidative bisulfite treated DNA. Bioinformatics.

[72] Smyth, G. K. Linear models and empirical bayes methods for assessing differential expression in microarray experiments. *Statistical applications in genetics and molecular biology* **3**, Article3, 10.2202/1544-6115.1027 (2004). 10.2202/1544-6115.1027 16646809

[73] Peters TJ (2015). De novo identification of differentially methylated regions in the human genome. Epigenetics & chromatin.

[74] Skvortsova K (2017). Comprehensive evaluation of genome-wide 5-hydroxymethylcytosine profiling approaches in human DNA. Epigenetics & chromatin.

[75] Chen EY (2013). Enrichr: interactive and collaborative HTML5 gene list enrichment analysis tool. BMC bioinformatics.

[76] Yu G, Wang LG, He QY (2015). ChIPseeker: an R/Bioconductor package for ChIP peak annotation, comparison and visualization. Bioinformatics.

[77] Cavalcante RG, Sartor M (2017). A. annotatr: genomic regions in context. Bioinformatics.

[78] Gu Z, Eils R, Schlesner M, Ishaque N (2018). EnrichedHeatmap: an R/Bioconductor package for comprehensive visualization of genomic signal associations. BMC genomics.

[79] Eisenberg E, Levanon EY (2013). Human housekeeping genes, revisited. Trends Genet..

[80] Hernandez-Munoz R (1984). Effects of adenosine on liver cell damage induced by carbon tetrachloride. Biochem. Pharmacol..

[81] Korinek M (2013). Quantification of homocysteine-related metabolites and the role of betaine-homocysteine S-methyltransferase in HepG2 cells. Biomed. Chromatogr..

