# Supplementary material for: Genome-wide 5-hydroxymethylcytosine (5hmC) emerges at early stage of in vitro differentiation of a putative hepatocyte progenitor
Source: Sci Rep. 2020 May 8;10:7822. doi: 10.1038/s41598-020-64700-2 (PMC7210258; doi:10.1038/s41598-020-64700-2)
Supplement: Supplementary file 1 — Supplementary Data. [file 41598_2020_64700_MOESM1_ESM.docx]

**Genome-wide 5-hydroxymethylcytosine (5hmC) emerges at early stage of *in vitro* differentiation of a putative hepatocyte progenitor**

Authors:

Jesús Rafael Rodríguez-Aguilera^1^

Szilvia Ecsedi^2^

Chloe Goldsmith^3^

Marie-Pierre Cros^4^

Mariana Domínguez-López^1^

Nuria Guerrero-Celis^1^

Rebeca Pérez-Cabeza de Vaca^1,5^

Isabelle Chemin^6^

Félix Recillas-Targa^7^

Victoria Chagoya de Sánchez^1^

Héctor Hernández-Vargas^4,8^

1. Department of Cellular Biology and Development, Instituto de Fisiología Celular, Universidad Nacional Autónoma de México (UNAM), Circuito Exterior s/n, Ciudad Universitaria, Coyoacán 04510, Cd. Mx., Mexico.
2. Institute of Biology Valrose (iBV), The National Center for Scientific Research (CNRS) - National Institute of Health and Medical Research (Inserm), Université Côte d’Azur, France.
3. Department of Immunity, Virus and Inflammation. Cancer Research Centre of Lyon (CRCL), Inserm U 1052, CNRS UMR 5286, Université de Lyon, Centre Léon Bérard, 28 rue Laennec, 69373 Lyon CEDEX 08, France.
4. Molecular Mechanisms and Biomarkers Group, International Agency for Research on Cancer (IARC), 150 Cours Albert Thomas, 69008 Lyon, France.
5. Division of Biomedical Research, Centro Médico Nacional “20 de noviembre”, ISSSTE,  San Lorenzo 502, Benito Juárez 03100, Cd. Mx., Mexico.
6. INSERM U1052, CNRS UMR5286, Centre de Recherche en Cancérologie de Lyon Université Claude Bernard, Lyon, France.
7. Department of Molecular Genetics, Instituto de Fisiología Celular, Universidad Nacional Autónoma de México (UNAM), Circuito Exterior s/n, Ciudad Universitaria, Coyoacán 04510, Cd. Mx., Mexico.
8. Department of Translational Research and Innovation. Centre Léon Bérard, 28 rue Laennec, 69373 Lyon CEDEX 08, France.

**Correspondence to:**

VCS: vchagoya@ifc.unam.mx

HHV: hector.hernandez-vargas@lyon.unicancer.fr

**Supplementary Data**

**
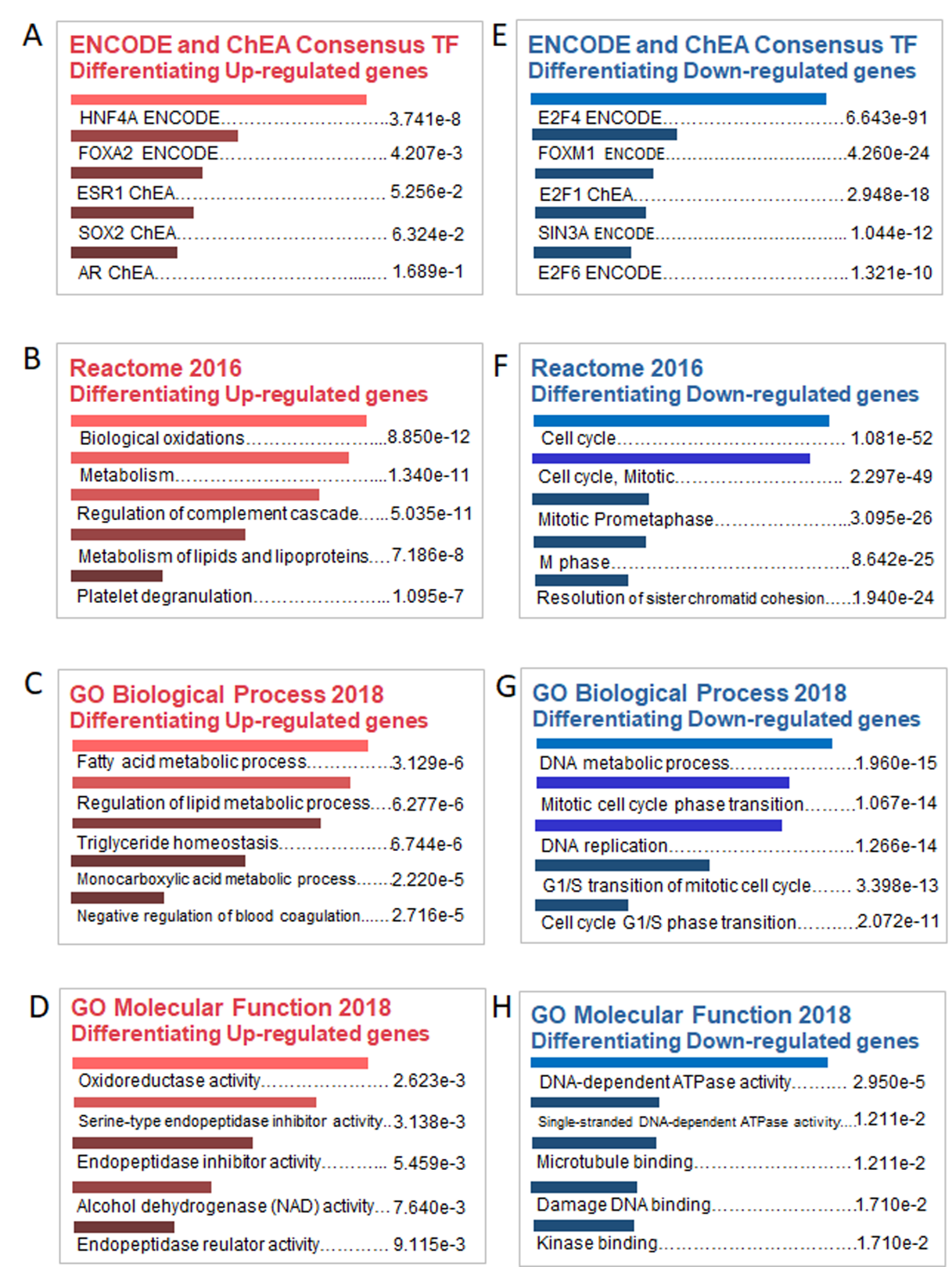
**

**Supplementary Figure S1. Gene ontologies from differentiating cells.** (A and E) Consensus transcription factors (TF) from Encyclopedia of DNA elements (ENCODE) and ChIP-X Enrichment Analysis (ChEA) of differentiating up-regulated genes and down regulated genes respectively. (B-D) Ontologies related with up-regulated genes from differentiating cells. (F-H) Ontologies related with down-regulated genes from differentiating cells. Data by EnrichR, number showed tissue-associated adjusted p-value.

**
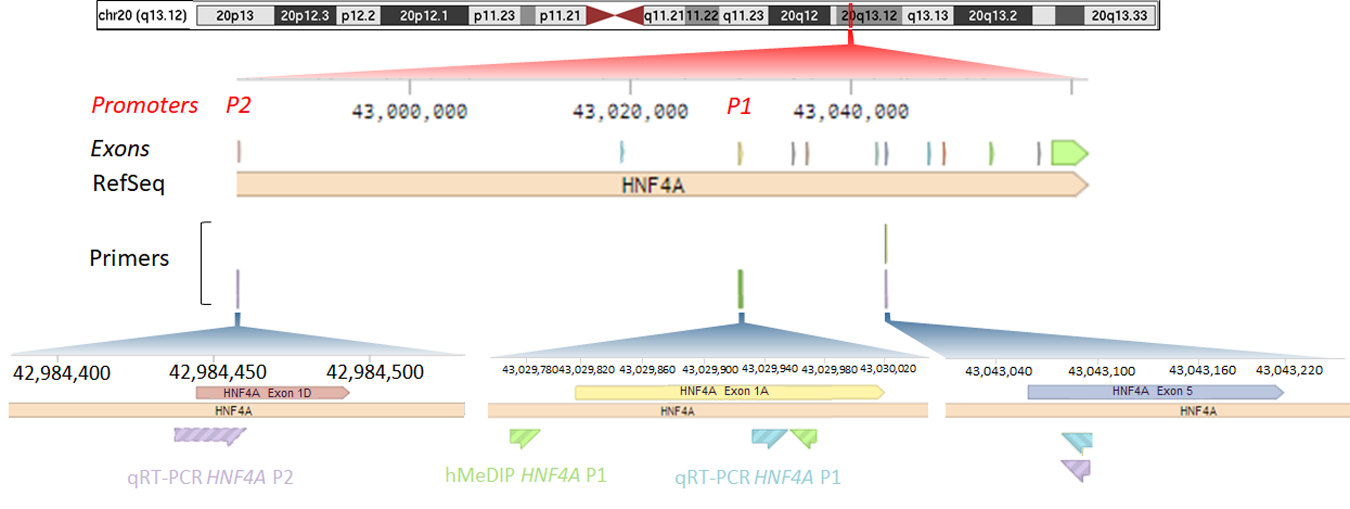
**

**Supplementary Figure S2. Analyzed regions on *HNF4A*.** Map shows a zoom in from chromosome 20 (q13.12) where *HNF4A locus* is located. Promoters P1 and P2 are indicated in red. *HNF4A* exons are denoted by colour boxes on *HNF4A* RefSeq (symbolized by pink pentagon). Primer pairs for qRT-PCR and hMeDIP are represented by colour boxes under *HNF4A* RefSeq. A zoom in from regions were primers were designed are shown, primer pairs to isoforms controlled by *HNF4A* P2 are denoted by half purple arrows, primer pairs to isoforms controlled by *HNF4A* P1 are represented by half blue arrows and primer pairs to hMeDIP on *HNF4A* P1 region are symbolized by half green arrows. Scheme armed with information from UCSC Genome Browser^35^ and constructed by Benchling platform (https://benchling.com).


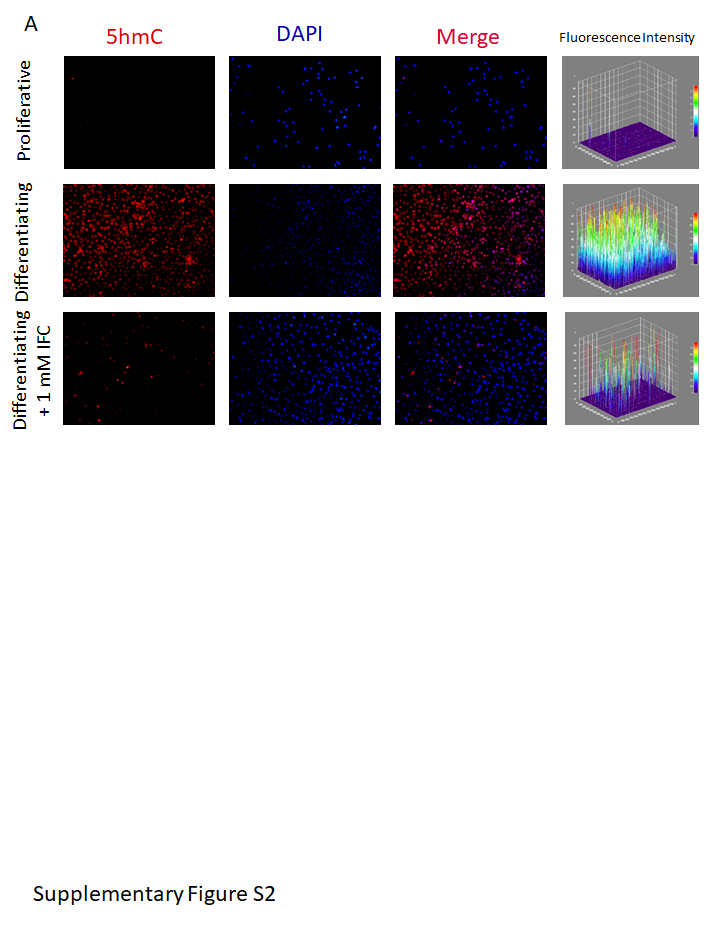


**Supplementary Figure S3. 5hmC appears in differentiating cells.** Immunofluorescence of 5hmC in proliferative (top panel), differentiating (middle panel) and differentiating + IFC-305 (bottom panel). Representative 20x images from 3 cultures/condition are shown, as well as measurement of fluorescence intensity.


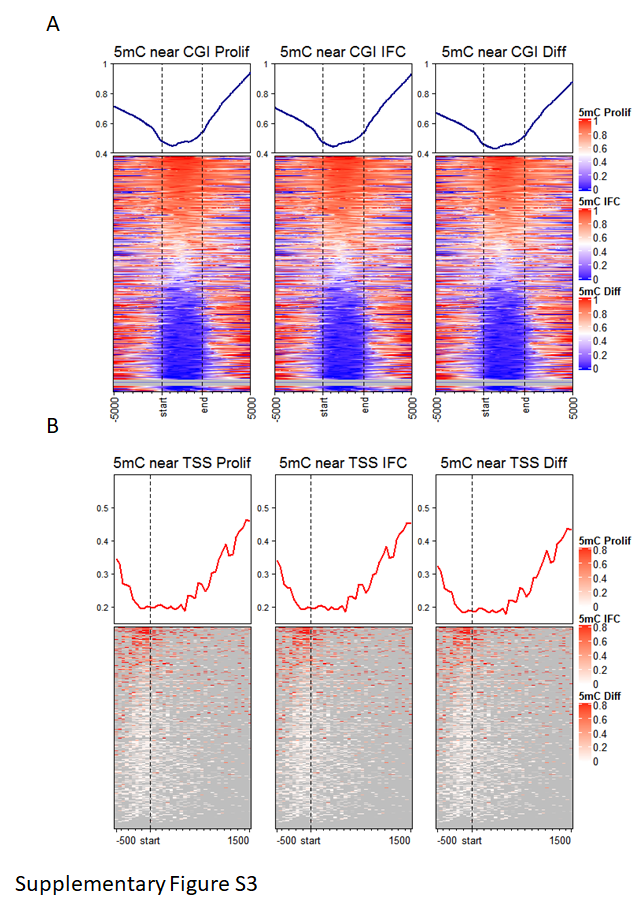


**Supplementary Figure S4. 5mC distribution on CpG islands and TSS during hepatocyte differentiation.** Global distribution of 5mC for one representative sample of each condition, according to CpG islands (A) and transcription start sites (B). In both cases, 5hmC levels are averaged across all hg19-annotated genomic regions. Two independent cultures were used for proliferative and differentiating cells, and three independent cultures for differentiating + 1 mM IFC-305.

**
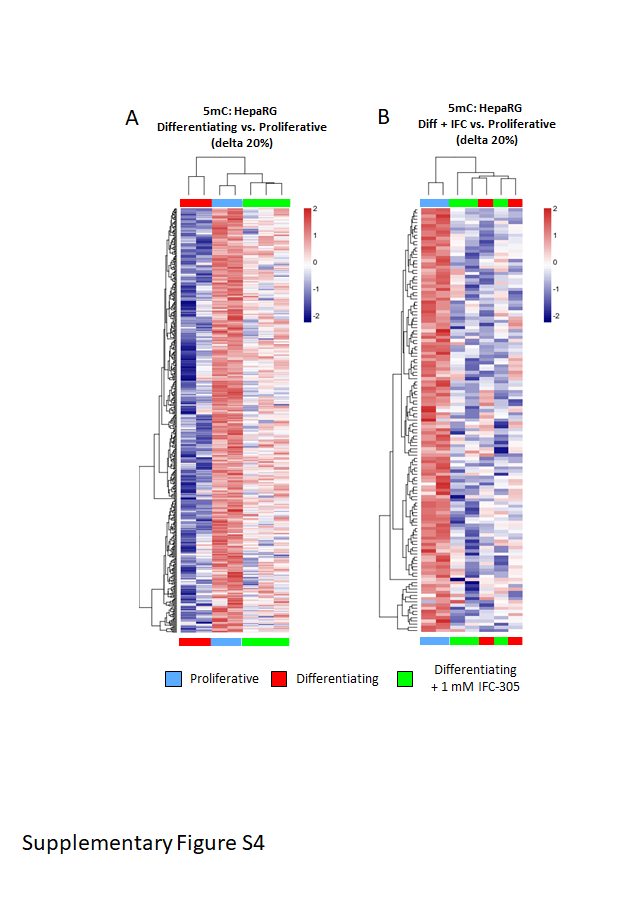
**

**Supplementary Figure S5. Differentiating process involves a genome-wide demethylation.** (A) Heatmap showing methylome comparison between differentiating and proliferative cells. (B) Heatmap showing methylome comparison between differentiating + IFC-305 and proliferative cells. Differentially methylated positions (DMPs) were filtered by the magnitude of change in methylation (delta beta) of at least 20% and p-adjusted value < 0.05. Experiments were performed in at least duplicates.

**
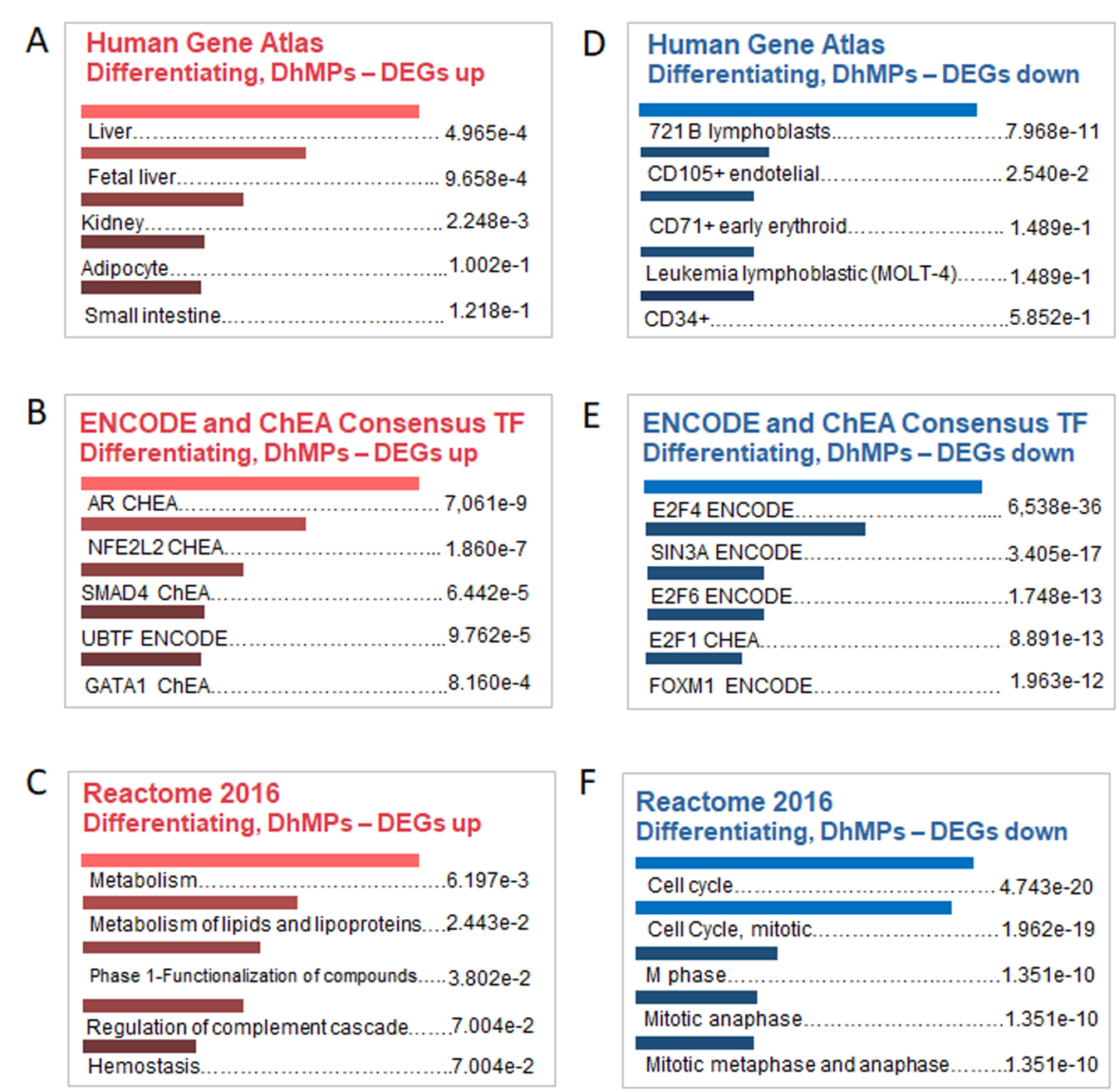
**

**Supplementary Figure S6. Ontologies from overlaps between DhMPs and DEGs.** (A) Cells/tissues associated with differentiating enriched 5hmC positions and up-regulated genes comparing Differentiating vs. Proliferative. (B) Consensus transcription factors (TF) from ENCODE ChEA of differentiating enriched 5hmC positions and up-regulated genes. (C) Ontologies related with differentiating enriched 5hmC positions and up-regulated genes. (D) Cells/tissues associated with differentiating enriched 5hmC positions and down-regulated genes comparing Differentiating vs. Proliferative. (E) Consensus transcription factors (TF) from ENCODE ChEA of differentiating enriched 5hmC positions and down-regulated genes. (F) Ontologies related with differentiating enriched 5hmC positions and down-regulated genes. Data from EnrichR, number showed tissue-associated adjusted p-value.


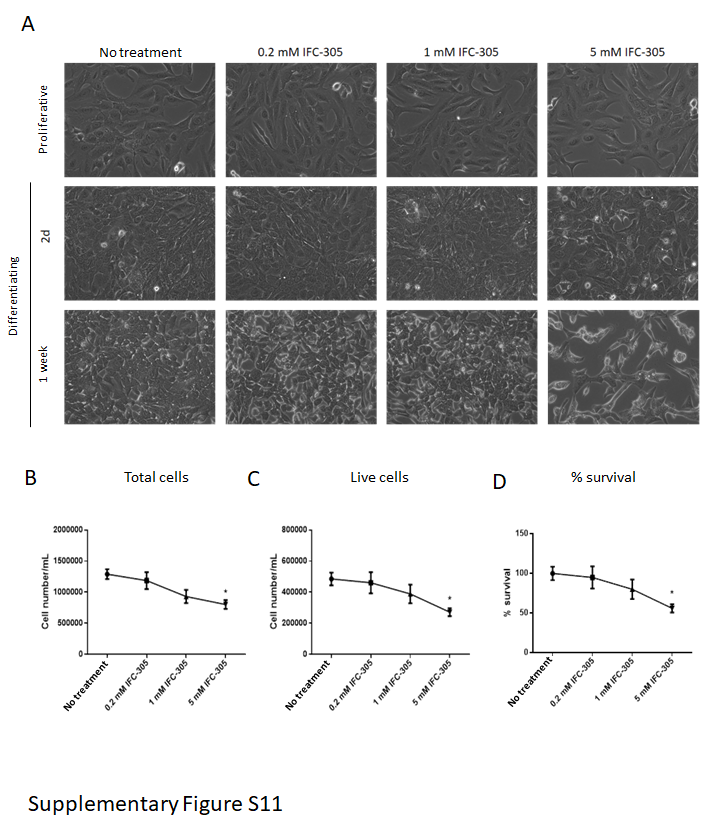


**Supplementary Figure S7. Low concentrated adenosine derivative did not alter HepaRG cell viability.** (A) HepaRG cell phenotype along 1 week of differentiation. 20x representative images of each group are shown (B) Total cell number. (C) Viable cell number. (D) Survival percentage. Data represent mean ± SEM of seven independent experiments. *Statistical difference (p < 0.05) compared with non-treated differentiating cells.
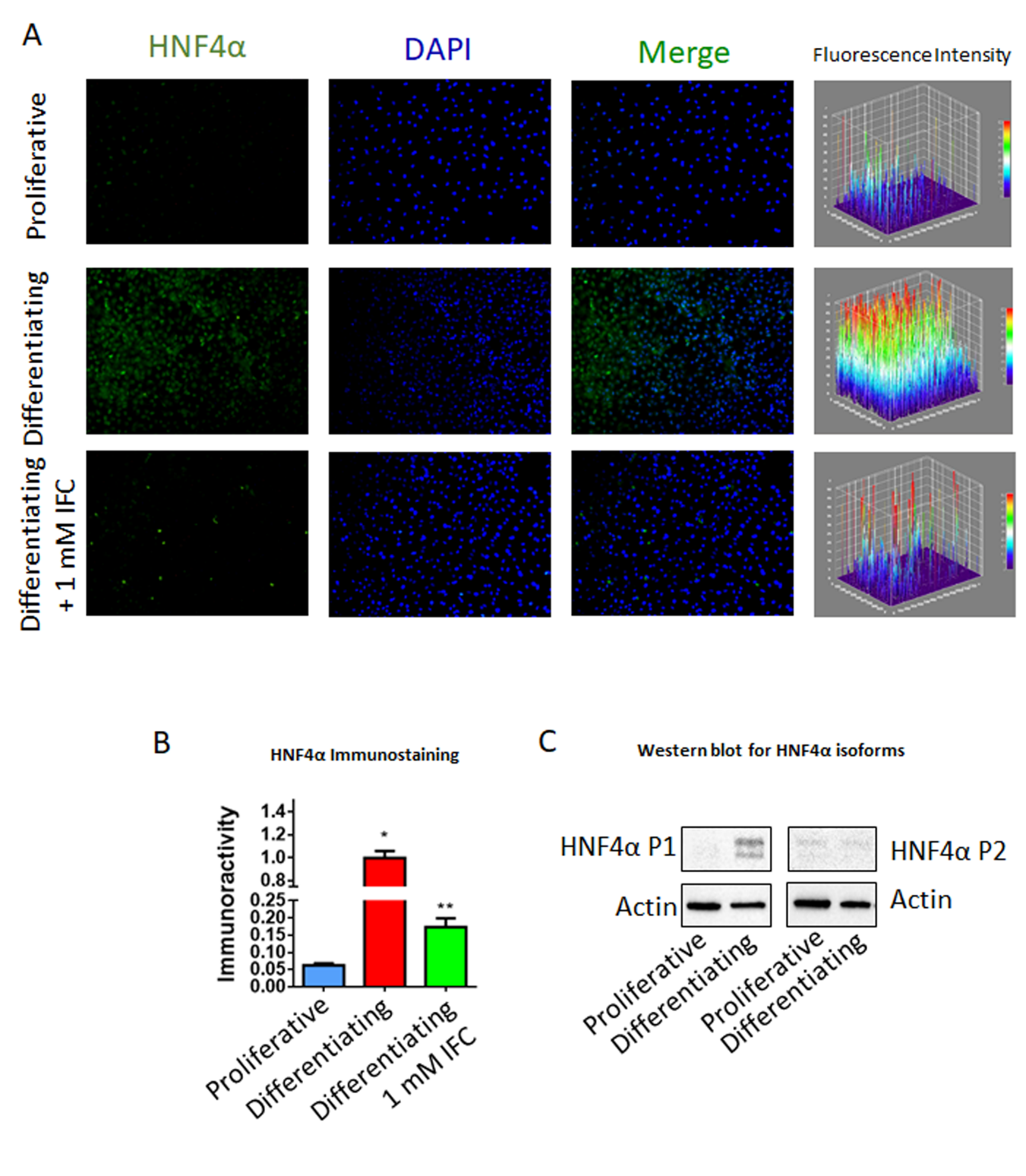


**Supplementary Figure S8. HNF4α increases in differentiating cells.** (A) Immunofluorescence of HNF4α in proliferative (top panel), differentiating (middle panel) and differentiating + IFC-305 (bottom panel). Representative images from 3 cultures/condition are shown. (B) Quantification of immunofluorescence HNF4α positive signal. Data represent mean ± SEM from 3 fields/group. *Statistical difference (p < 0.05) when compared with proliferative cells. **Statistical difference (p < 0.05) compared with differentiating cells. (C) Western blots with HNF4α isoform-specific antibodies on total proteins from proliferative and differentiating HepaRG cells. Actin was used as housekeeping protein.

**
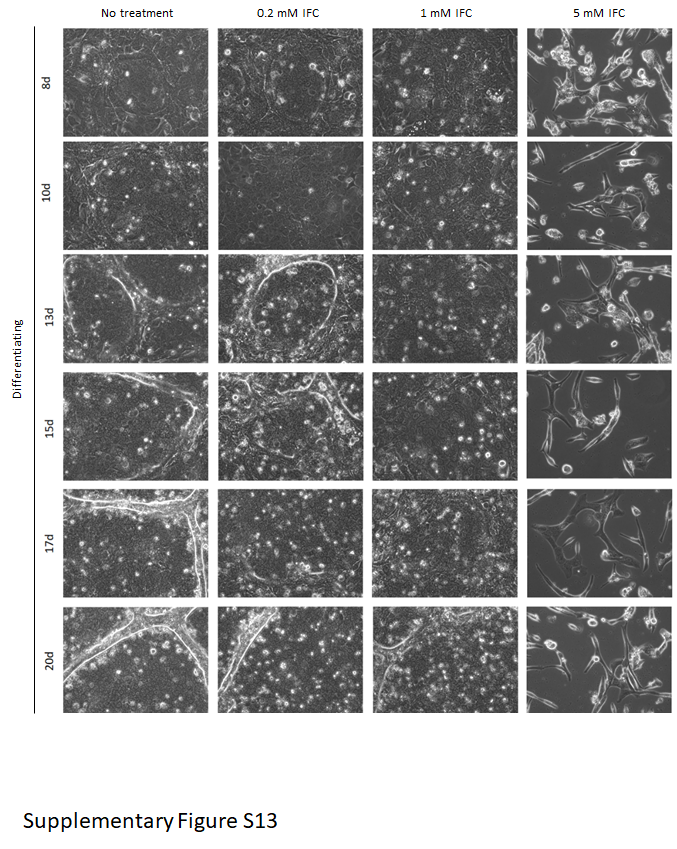
**

**Supplementary Figure S9. 1mM IFC-305 differentiating exposed cells presents a less differentiated phenotype.** 20 days time-lapse images showing HepaRG phenotype during differentiation with an increasing gradient of IFC-305. Representative images of 7 independent experiments. Representative 20x magnification images from seven independent cultures are shown.


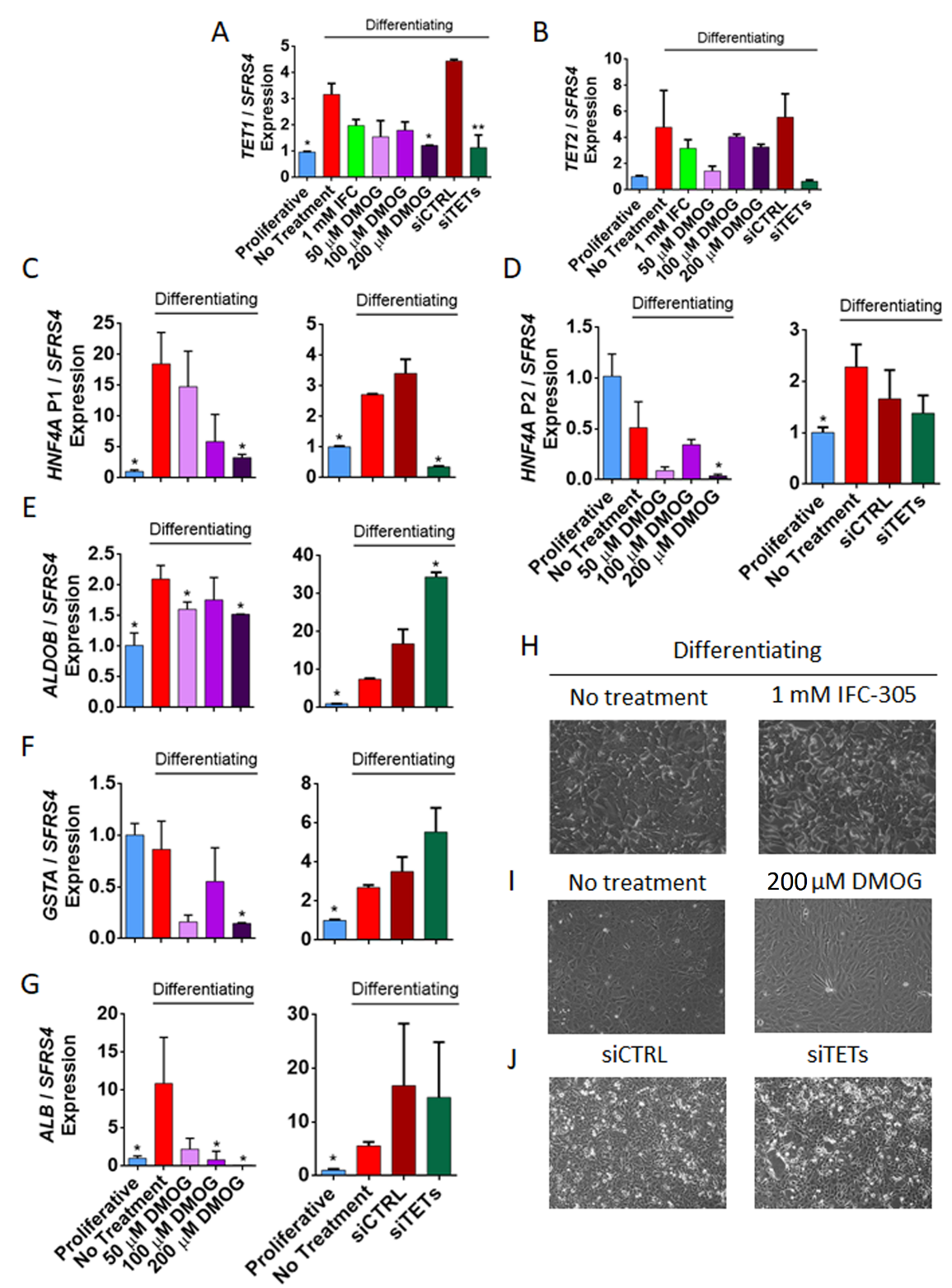


**Supplementary Figure S10. Impaired hepatocyte marker expression upon TETs transcription diminishment**. *TET1* (A) and *TET2* (B) expression after IFC-305, DMOG or siTETs exposed HepaRG cells during 1 week of differentiation; (C-G) Hepatocyte markers modified expression in response to DMOG or siTETs exposure, during 1 week of HepaRG differentiation. Data represent mean ± SEM 3 independent cultures/condition. *Statistical difference (p < 0.05) when compared with differentiating non-treated cells. **Statistical difference (p < 0.05) when compared with differentiating siCTRL exposure. HepaRG cells phenotype between non treated cells and 1 mM IFC-305 (H) or 200 μM DMOG (I), and between siCTRL and siTETs (J) at 1 week of differentiation; representative 20x magnification images from 3 independent cultures are shown.

**
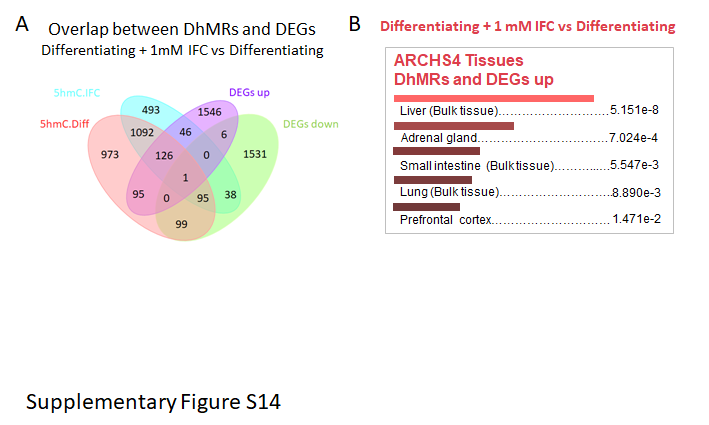
**

**Supplementary Figure S11. Some differential hydroxymethylated regions in IFC-305 treated differentiating cells, were related with differential overexpressed genes.** (A) Comparison between differential 5-hydroxymethylated regions (DhMRs) in IFC-305 exposed cells and DEGs in differentiating cells. (B) Ontologies related with differentiating + IFC-305 enriched 5hmC regions and up-regulated genes in differentiating cells. Data from EnrichR, number showed tissue-associated adjusted p-value.

**
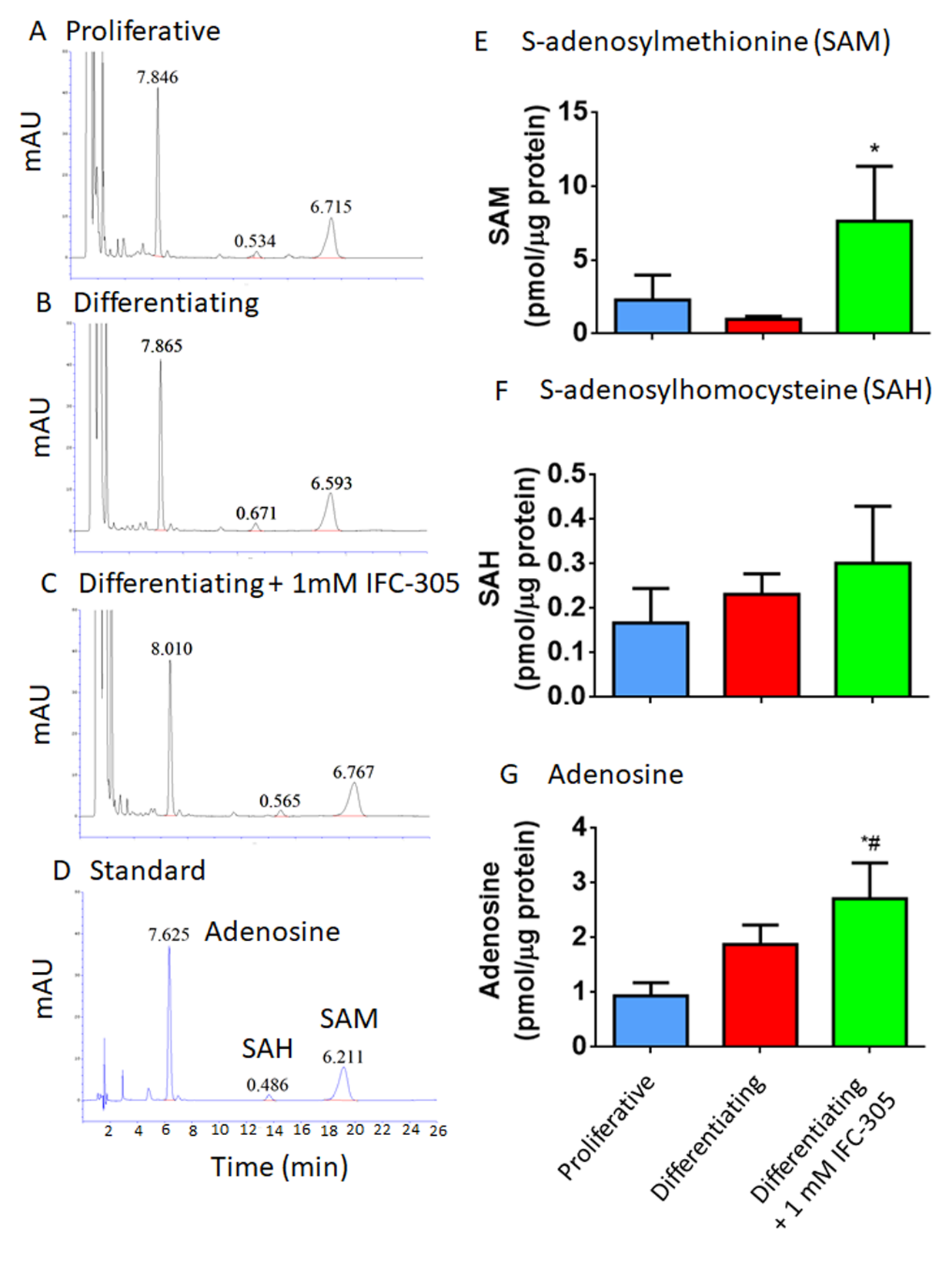
**

**Supplementary Figure S12.** **S-adenosylmethionine trends to decrease with HepaRG differentiation.** (A-D) Representative chromatograms identifying adenosine, S-adenosylhomocysteine (SAH) and S-adenosylmethionine (SAM) through HPLC in analyzed conditions, numbers indicate area under curve, first 26 min separation runtime are shown. (E-G) Quantification of each analysis. Data represent mean ± SEM of 4 cultures/condition. *Statistical difference (p < 0.05) when compared with differentiating non-treated cells. *^#^Statistical difference (p < 0.05) when compared with proliferative cells.


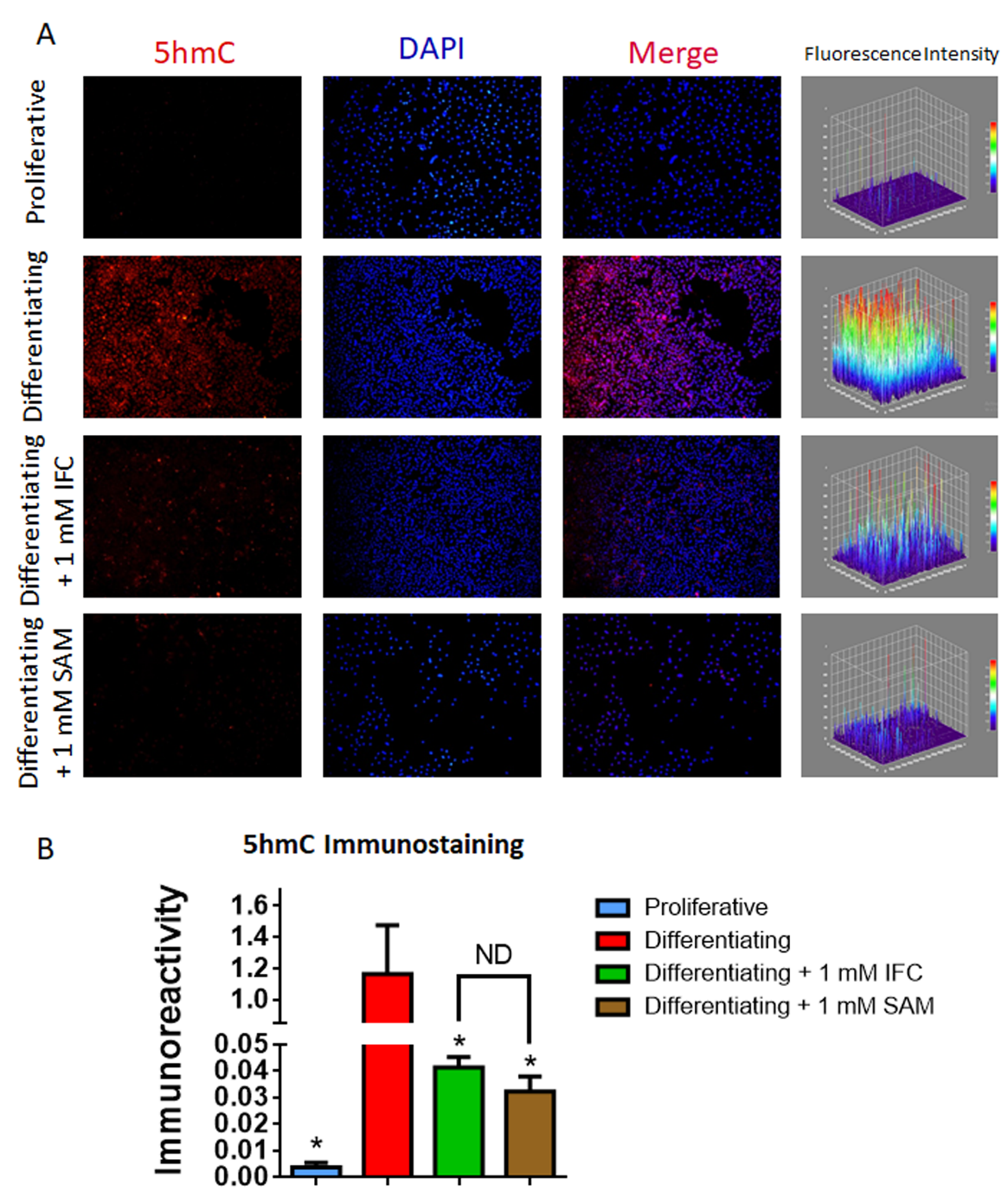


**Supplementary Figure S13. Adenosine derivative and S-adenosylmethionine reduce 5hmC enrichment at one week of HepaRG differentiation.** (A) Immunofluorescence of 5hmC in proliferative (top panel), differentiating (up-middle panel), differentiating + IFC-305 (down-middle panel), and differentiating + SAM (bottom panel). Representative 10x images of 3 cultures/condition are shown, as well as measurement of fluorescence intensity. (B) Quantification barplots of 5hmC signal are based on mean ± SEM from 3 fields per group. *Statistical difference (p < 0.05) compared with differentiating cells. ND: non-difference comparing differentiating + 1 mM IFC and differentiating + 1 mM SAM.


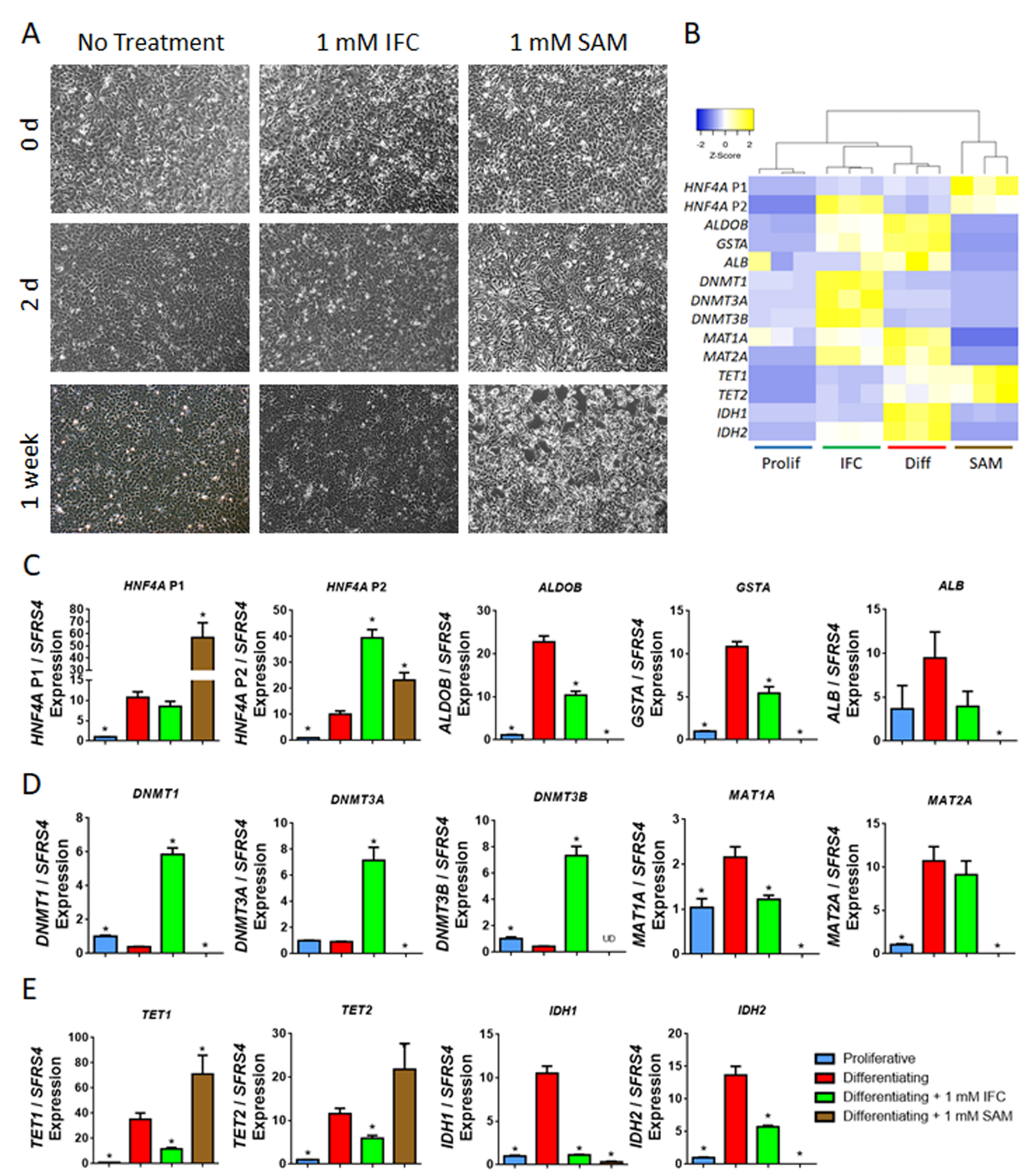


**Supplementary Figure S14. Impaired expression of hepatocytes markers, DNA methylation pathway and cytosine oxidation pathway upon adenosine derivative and S-adenosylmethionine HepaRG treatment.** (A) HepaRG cell phenotype along 1 week of differentiation, representative 10x images of each group are showed. (B) Heatmap represents differential expression of analysed genes and was constructed by Heatmapper platform (http://heatmapper.ca/). (C) Hepatocyte differentiation markers expression in response to different treatments at 1 week of differentiation. (D) DNA methyltransferases and methionine adenosyltransferases expression after 1 week of exposure during differentiation process. (E) TETs dioxigenases and isocytrate dehidrogenases expression after 1 week of differentiation and effect of IFC-305 and SAM exposure. Data represent mean ± SEM 3 independent cultures/condition. *Statistical difference (p < 0.05) when compared with differentiating non-treated cells. UD: undetectable.


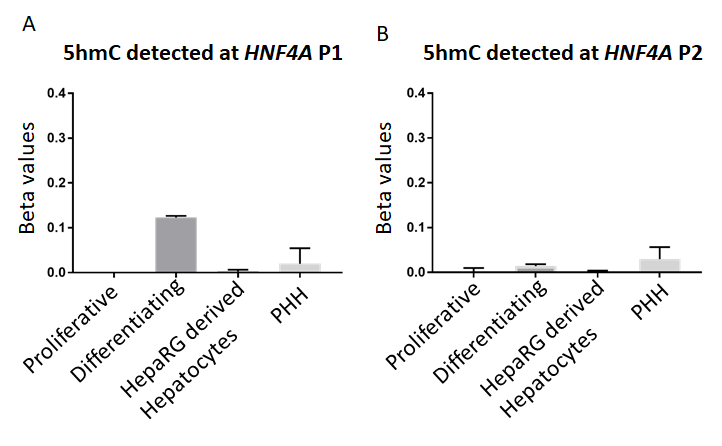


**Supplementary Figure S15.** 5hmC quantification by oxBS-qMSP, in HNF4A promoters P1 (A) and P2 (B). PHH= primary human hepatocytes.


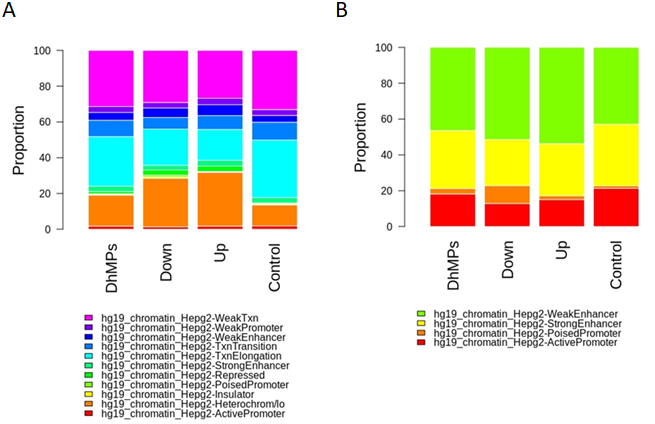


**Supplementary Figure S16. Distribution of DhMPs in Differentiating vs Proliferative HepaRG cells, relative to histone marks.** Genomic regions exhibiting differential 5hmC were annotated using ChromHMM features. The annotation determines several different chromatin states (A) and we emphasized the corresponding to H3K4me1/H3K27ac chromatin marks (B). The figure shows all 5hmC genes DhMPs, up and downregulated genes and control/housekeeping genes.

**Supplementary Table S1.** List of primers used for quantitative RT-PCR.

| **Region** | **Sequence** |
| --- | --- |
| SFRS4^26^ | Fw (5'-GGCTACGGGAAGATCCTGGA-3') |
|  | Rv (5'-TGCATCACGCAGATCATCAA-3') |
| *ALDOB*^26^ | Fw (5'-TGTTCACACGGGTTCTTCTG-3') |
|  | Rv (5'-CGGGCATTGGACCCTAGTA-3') |
| *GSTA*^26^ | Fw (5'-GGGAGAGAACTATTGAGAGGAACA-3') |
|  | Rv (5'-TTAAACGCTGTCACCGTCCT-3') |
| *ALB*^26^ | Fw (5'-GCTTATTCCAGGGGTGTGTTT-3') |
|  | Rv (5'-CTGAGCAAAGGCAATCAACA-3') |
| *HNF4A* P1^26^ | Fw (5'-ATGGACATGGCCGACTACAGTGCT-3') |
|  | Rv (5'-CGAATGTCGCCGTTGATC-3') |
| *HNF4A* P2^26^ | Fw (5'-CTTGGCCATGGTCAGCGTGAACG-3') |
|  | Rv (5'-CGAATGTCGCCGTTGATC-3') |
| *TET1^26^* | Fw (5'-GCTATACACAGAGCTCACAG-3') |
|  | Rv (5'-GCCAAAAGAGAATGAAGCTCC-3') |
| *TET2^26^* | Fw (5'-CTTTCCTCCCTGGAGAACAGCTC-3') |
|  | Rv (5'-TGCTGGGACTGCTGCATGACT-3') |
| *IDH1* | Fw (5'-CGGAACCCAAAAGGTGACAT-3') |
|  | Rv (5'-TGGCAACACCACCACCTTCT-3') |
| *IDH2* | Fw (5'-CACGGCCTCAGCAATGTG-3') |
|  | Rv (5'-TCGAGGAAGTCCGTGGTGTT-3') |
| *DNMT1* | Fw (5'-GATGTGGCGTCTGTGAGGT-3') |
|  | Rv (5'-CCTTGCAGGCTTTACATTTCC-3') |
| *DNMT3A* | Fw (5'-CCTGAAGCCTCAAGAGCAGT-3') |
|  | Rv (5'-TGGTCTCCTTCTGTTCTTTGC-3') |
| *DNMT3B* | Fw (5'-CAAATGGCTTCAGATGTTGC-3') |
|  | Rv (5'-TCCTGCCACAAGACAAACAG-3') |
| *MAT1A* | Fw (5'-GGGCAGGAGATCAGGGTTTG-3') |
|  | Rv (5'-GCCCTGATGACTTGCTCCTT-3') |
| *MAT2A* | Fw (5'-CTACGAGTAGAACGCTGTCCG-3') |
|  | Rv (5'-CCAGTTTTAGCAACAGTTTCACA-3') |

**Supplementary Table S2.** List of primers used for hMedIP.

| **Region** | **Sequence** | **Location (hg19)** |
| --- | --- | --- |
| *SFRS4* P | Fw (5'-CCTCTTTCGCCCTCCTCA-3') | chr1:29508139- |
|  | Rv (5′-TGTAGGCTGGGTCCAGTG-3′) | 29508338 |
| *HNF4A* P1^26^ | Fw (5′-ATCTTCCCAGAGGACGGTTT-3′) | chr20:43029881- |
|  | Rv (5′-TGTAGGCTGGGTCCAGTG-3′) | 43030082 |
| *TCHP* | Fw (5'-GCCAGGTTCTCTGCATCAT-3') | chr12:110352469- |
|  | Rv (5'-ATGGCATGTACCTCGTAGAAAG-3') | 110352877 |
| *RAB7A* | Fw (5'-TCTGAGTCCTGGTGGTAGTT-3') | chr3:128483808- |
|  | Rv (5'-GTTGTGGGTAGCAGAGACATT-3') | 128484074 |

**Supplementary Table S3.** List of primers used for Hydroxymethylation by oxidative bisulfite and quantitative methyl-specific PCR

| **Region** | **Sequence** |
| --- | --- |
|  | Methylated |
| *HNF4A* P1 | Fw (5'-TGAGTTAAGGGTTAAATGAGTGTTC-3') |
|  | Rv (5'-CGCCGATAAATAAACTAAACGAA-3') |
| *HNF4A* P2 | Fw (5'-GAGATTTAAAATTGAGATAAAAGAAACG-3') |
|  | Rv (5'-CAAAATCATCCTATCTAACGTCT-3') |
|  | Unmethylated |
| *HNF4A* P1 | Fw (5'-GTGAGTTAAGGGTTAAATGAGTGTTT-3') |
|  | Rv (5'-CCCACCAATAAATAAACTAAACAAA-3') |
| *HNF4A* P2 | Fw (5'-ATTTAAAATTGAGATAAAAGAAATGGG-3') |
|  | Rv (5'-AACAAAATCATCCTATCTAACATCT-3') |
